# Ets-2 Propagates IL-6 Trans-Signaling Mediated Osteoclast-Like Changes in Human Rheumatoid Arthritis Synovial Fibroblast

**DOI:** 10.3389/fimmu.2021.746503

**Published:** 2021-11-02

**Authors:** Anil K. Singh, Mahamudul Haque, Bhanupriya Madarampalli, Yuanyuan Shi, Benjamin J. Wildman, Abdul Basit, Sadik A. Khuder, Bhagwat Prasad, Quamarul Hassan, Madhu M. Ouseph, Salahuddin Ahmed

**Affiliations:** ^1^ Department of Pharmaceutical Sciences, Washington State University College of Pharmacy, Spokane, WA, United States; ^2^ Department of Pharmaceutics, University of Washington School of Medicine, Seattle, WA, United States; ^3^ Department of Oral and Maxillofacial Surgery, School of Dentistry, University of Alabama at Birmingham, Birmingham, AL, United States; ^4^ Department of Medicine and Public Health, University of Toledo, Toledo, OH, United States; ^5^ Department of Pathology and Laboratory Medicine, Weill Cornell Medical College, New York, NY, United States; ^6^ Division of Rheumatology, University of Washington School of Medicine, Seattle, WA, United States

**Keywords:** osteoclast, interleukin-6, synovial fibroblasts, rheumatoid arthritis, reprogramming

## Abstract

Rheumatoid arthritis synovial fibroblasts (RASFs) contribute to synovial inflammation and bone destruction by producing a pleiotropic cytokine interleukin-6 (IL-6). However, the molecular mechanisms through which IL-6 propels RASFs to contribute to bone loss are not fully understood. In the present study, we investigated the effect of IL-6 and IL-6 receptor (IL-6/IL-6R)-induced trans-signaling in human RASFs. IL-6 trans-signaling caused a significant increase in tartrate-resistant acid phosphatase (TRAP)-positive staining in RASFs and enhanced pit formation by ~3-fold in the osteogenic surface *in vitro*. IL-6/IL-6R caused dose-dependent increase in expression and nuclear translocation of transcription factor Ets2, which correlated with the expression of osteoclast-specific signature proteins RANKL, cathepsin B (CTSB), and cathepsin K (CTSK) in RASFs. Chromatin immunoprecipitation (ChIP) analysis of *CTSB* and *CTSK* promoters showed direct Ets2 binding and transcriptional activation upon IL-6/IL-6R stimulation. Knockdown of Ets2 significantly inhibited IL-6/IL-6R-induced RANKL, CTSB, and CTSK expression and TRAP staining in RASFs and suppressed markers of RASF invasive phenotype such as Thy1 and podoplanin (PDPN). Mass spectrometry analysis of the secretome identified 113 proteins produced by RASFs uniquely in response to IL-6/IL-6R that bioinformatically predicted its impact on metabolic reprogramming towards an osteoclast-like phenotype. These findings identified the role of Ets2 in IL-6 trans-signaling induced molecular reprogramming of RASFs to osteoclast-like cells and may contribute to RASF heterogeneity.

## Introduction

Rheumatoid arthritis (RA) is a chronic autoimmune disease characterized by synovial inflammation, hyperplasia, and pannus formation, leading to progressive joint destruction. RA synovial fibroblasts (RASFs) contribute to bone and cartilage degradation by producing numerous inflammatory cytokines and matrix metalloproteinases (1, 2). High osteoclast-like activity in the joints is a major contributing factor to bone deformities and progressive bone loss in RA patients (3, 4). Previous studies suggest that SFs could be converted to osteoblast-like cells by induced cellular reprogramming (5). Using a heterogenous cell population commonly present in RA synovial tissue, Takayangi et al. (6, 7) reported that RASFs could be differentiated into osteoclast-like cells by chronic stimulation with a low dose of macrophage colony stimulating factor (M-CSF) and receptor activator nuclear factor-κB ligand (RANKL), suggesting the role of SFs in modulating bone health by tilting balance towards osteolytic function. Interleukin-6 (IL-6) is a proinflammatory cytokine produced by RASFs that contributes to osteoclastogenesis (8) by further activating RANKL expression (9), and, as we show herein, predisposes RASFs to osteoclast-like signaling and/or differentiation. However, the mechanism by which IL-6 independently activates the signaling pathways and/or crosstalks with M-CSF and RANKL in RASFs to stimulate and maintain the osteoclast-like reprogramming in the synovial microenvironment remains unclear.

Osteoclasts are traditionally formed by the fusion of mononuclear cells (monocytes) that stay in a multinuclear state. It is mainly a CD14^+^ mononuclear subtype that can differentiate into osteoclasts (10, 11). The formation of osteoclasts depends on RANKL signaling *via* the RANK receptor, whereas osteoprotegerin (OPG), also known as TNFRSF11B, is a decoy ligand for RANK produced by various cells and an endogenous inhibitor for RANKL binding to the RANK receptor (12, 13). When RANKL signaling predominates OPG signaling, it induces differentiation of osteoclast precursor cells to mature osteoclasts (13). Exogenous M-CSF makes cells resistant to apoptosis and, thereby, promotes osteoclast proliferation (14–16). Since SFs express M-CSF, RANK, and OPG, these cells possess the predeterminants to undergo metabolic reprogramming to osteoclast-like cells and may independently contribute or accelerate the bone resorption in the diseased joints.

Current understanding of IL-6 biology suggests that it contributes to bone destruction by accelerating osteoclastogenesis in RA (17), with IL-6 trans-signaling contributing to bone formation and osteoclastogenesis (18). However, there are several gaps in this understanding, such as the potential role of IL-6 in forcing a phenotypic switch in RASFs to osteoclast-like cells, the key signaling mediators involved in this process, and if the effect of IL-6 trans-signaling on metabolic reprogramming is independent or in crosstalk with M-CSF/RANKL signaling. The present study was undertaken to identify the novel mediators influenced by IL-6 signaling to accelerate the phenotypic conversion of RASFs to osteoclast-like cells and to elucidate the effects of Ets2 transcription factor in bone destruction in RA.

## Materials and Methods

Detailed information about antibodies and reagents, Western immunoblotting, ELISA, and *in vitro* scratch test are provided in the *Materials and Methods* section of the **Supplementary Information (SI)**.

### Isolation and Culture of Human Nondiseased SFs and RASFs

The deidentified nondiseased (NL) and RA synovial tissues (RASTs) were procured under a protocol approved by the Washington State University IRB (IRB#14696) from the Cooperative Human Tissue Network (Columbus, OH) and National Disease Research Interchange (Philadelphia, PA). STs from RA patients were obtained from joint surgery or synovectomy according to an Institutional Review Board (IRB)-approved protocol and in compliance with the Declaration of Helsinki. NLSTs were obtained from autopsy or amputation. Disease RA tissue was digested in dispase, collagenase, and DNase before being seeded in 72 cm^2^ flasks.

Cells were grown in RPMI 1640 medium supplemented with 15% fetal bovine serum (FBS, Invitrogen, Waltham, MA, USA), 5,000 U/ml penicillin, 5 mg/ml streptomycin from Sigma (St. Louis, MO, USA), and 10 µg/ml gentamicin (Invitrogen, Waltham, MA, USA). Upon confluency (>85%), cells were passaged with brief trypsinization. Experiments were done using cells passed at least four to five times to ensure a pure fibroblast population. Similarly, we also established primary human synovial fibroblasts from nondiseased (NLSFs) tissues under an IRB-approved protocol.

### Cell Culture and Stimulation

Human primary NLSF and RASF cells were maintained in RPMI 1640 culture media, with 10% FBS and antibiotics. For chronic exposure of cytokines ranging 12 days of exposure, 2 ng/ml M-CSF+10 ng/ml RANKL were used with or without a combination of 100 ng/ml IL-6 along with 100 ng/ml IL-6Rα. For acute exposure, cells were starved overnight in plain RPMI 1640, followed by stimulation cytokines. Acute treatment of cytokines ranging between 30 min and 4 h using 25 ng/ml MCSF+50 ng/ml RANKL was used with or without combination 100 ng/ml IL-6 along with 100 ng/ml IL-6R experiments. For chronic exposure of cells, RPMI 1640 with 10% FBS in combination with cytokines was changed every 3 days. On day 11 of differentiation, media without FBS was replenished for an additional 24 h before termination of the experiment. Supernatant and whole-cell extracts were prepared accordingly for further experiments. Likewise, we isolated bone marrow cells from 8-week-old C57BL/6 and allowed them to differentiate using 25 ng/ml M-CSF+10 ng/ml RANKL for 14 days with or without 100 ng/ml IL-6 or 100 ng/ml IL-6+100 ng/ml IL-6R (IL-6/IL-6R) for bone marrow macrophage-derived osteoclast studies. Human PBMCs were obtained from a commercial source (Zen-Bio, Inc., Research Triangle, NC, USA).

For the inhibitor experiments, overnight-starved RASF cells were subjected to 2 h pretreatment with 5 µM tofacitinib or 3 µg/ml anti-IL-6 antibody followed by M-CSF+RANKL and/or IL-6/IL-6R stimulation for 30 min for Western blotting. TRAP staining was performed on RASFs based on manufacturer instructions from PMC-AK04F-COS (CosmoBio, Carlsbad, CA, USA).

### Chromatin Immunoprecipitation Assay

Chromatin immunoprecipitation (ChIP) was performed for Ets2 as described previously (19). Human RASFs from three different donors were subjected to differentiation by M-CSF+RANKL with or without IL-6+IL-6R combination for 12 days. Detailed protocol is provided in the *SI Materials and Methods.* The list of primer pairs used for the ChIP assay has been provided in *SI* (**Supplementary Table S1**).

### Untargeted Proteomics of RASFs

The untargeted proteomics analysis was conducted on an Orbitrap Fusion Lumos mass spectrometer (Thermo Fisher Scientific, Waltham, MA, USA) equipped with a Nano-Acquity UPLC system (Waters, Milford, MA, United States) and in-house developed nanospray ionization source at the University of Washington Proteomics Center, Seattle, WA. A detailed method is provided in the *SI Material and Methods* file. Candidates from IL-6+IL-6R treatment cohorts were also subjected to Metascape and or Ingenuity Pathways Analysis-based (IPA-Qiagen, Hilden, Germany) gene ontology prediction. The mass spectrometry proteomics data have been deposited to the ProteomeXchange Consortium *via* the PRIDE partner repository with the dataset identifier PXD028374.

### *In Vitro* Pit Formation Assay

Human RASFs (2 × 10^4^) were seeded in calcium phosphate-coated 24-well plates (CSR-BRA-24KIT, CosmoBio, Carlsbad, CA, USA) and allowed to reach ∼85% confluency. Cells were differentiated under 2 ng/ml M-CSF+10 ng/ml RANKL with and without combination of 100 ng/ml IL-6+100 ng/ml IL-6R for 12 days. Every 3 days, media were replaced with fresh cytokines. After 12 days of differentiation, plates were washed with 10% sodium hypochlorite for 45 min, followed by an extensive wash using deionized water. Plates were air-dried, followed by staining with 1% toluidine blue staining for an additional 5 min. Plates were rewashed and air-dried. Each well of toluidine blue stain plates were photographed using a Zeiss microscope. Pit areas (three independent pits from each triplicate well) were calculated using ImageJ software (NIH, Bethesda, MD, United States). The total cumulative pit area was divided by the whole field and reported as a percentage of pit area per field.

### Statistical Analysis

Statistical analysis of multiple comparisons between control *versus* treatment group means was performed using a one-way ANOVA followed by Dunnett’s test or using Tukey’s multiple comparison test when comparing multiple treatment groups to each other. All tests assumed normal distribution where *p* < 0.05 was considered significant.

## Results

### IL-6 Trans-Signaling Enhances Osteoclast-Like Features in Human RASFs *In Vitro*

**Figure 1:**
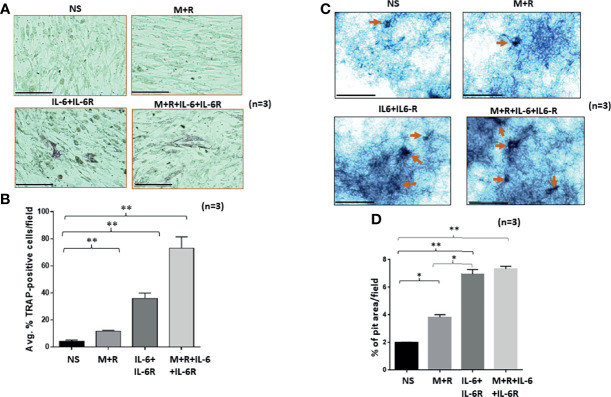
Human RASFs in the presence of IL-6/IL-6R exhibit enhanced TRAP-positive staining and transform to an invasive phenotype. **(A)** RASFs grown with M-CSF (2 ng/ml)+ RANKL (10 ng/ml) (M+R) alone or with IL-6 and IL-6R (100 ng/ml each) for 12 days show an increased number of TRAP-positive RASFs compared with M-CSF/RANKL-differentiated and nonstimulated (NS) cell population. **(B)** IL-6+IL-6R treatment found to be almost 40% TRAP-positive cells which is more synergetic in combination with MCSF+RANKL where 70% of RASF cells are TRAP positive. **(C)** RASFs grown on calcium phosphate plates with M-CSF+ANKL alone or with IL-6+IL-6R for 12 days shows the increased pit formation on calcium phosphate-coated plates. **(D)** An average of three independent field quantifications of pits formed by RASFs from *n* = 3 patient donor samples reveal a threefold increase in the number of pits formed in calcium phosphate-coated plates when RASFs were stimulated with IL-6/IL-6R alone or in combination with M-CSF/RANKL as compared with nonstimulated RASFs, presented as mean ± SEM. ^*^ *p* < 0.05; ^**^ *p* < 0.01. Bar scale for images acquired is 150 µm.

To understand the development of TRAP-positive RASFs that indicate osteoclast-like phenotype, human RASFs treated under different conditions for 12 days were washed with ice-cold PBS, fixed immediately, and stained using a TRAP substrate. Analysis of the stained area showed that IL-6/IL-6R increased the number of TRAP-positive cells in cultured conditions more than M-CSF/RANKL, the known drivers of osteoclastogenesis (**Figures 1A, B**). To confirm this observation, we differentiated bone marrow-derived macrophage (BMDM) using M-CSF/RANKL or treated in combination with IL-6 for 14 days. Upon differentiation, IL-6 markedly increased in the number of TRAP-positive BMDMs compared with M-CSF/RANKL treatment (**Supplementary Figure S1**). To confirm this phenotypic switch of RASFs to osteoclast-like cells also influenced their function, we performed the *in vitro* pit formation assay using calcium phosphate-coated plates. Evaluation of the pit areas showed that RASFs treated with chronic IL-6/IL-6R caused a significantly higher erosion of calcium phosphate from the plates independent of the osteoclastic cytokines M-CSF/RANKL (**Figure 1C**). Quantitative analysis showed that the percentage of pit area formed were significantly increased by ~2-fold in IL-6/IL-6R-treated samples compared with M-CSF/RANKL, and no further increase in the percentage of pit area formed was observed with M-CSF/RANKL plus IL-6/IL-6R treatment (**Figure 1D**; *p* < 0.01; *n* = 3), suggesting that IL-6 trans-signaling can independently be a major factor in RASF-induced bone loss. Furthermore, our FACS analysis identified the lack of CD14+ cell population in RASF culture preparations used in the study, suggesting the pure synovial fibroblast lineage of the cells used (**Supplementary Figure S2**).

### Ets2 Mediates IL-6 Trans-Signaling Induced RASF Reprogramming to Osteoclast-Like Cells

**Figure 2:**
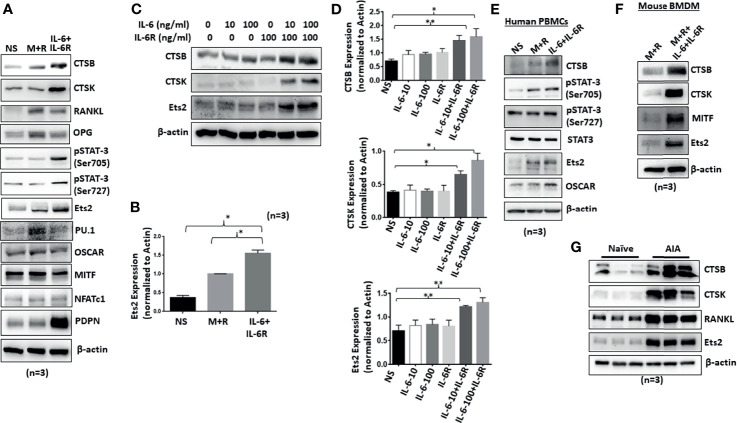
Chronic exposure of IL-6/IL-6R induces cathepsin K, cathepsin B, and Ets2 expression in RASFs. **(A)** Western blot analysis of whole cell extracts prepared from human RASFs grown in the presence of IL-6+IL-6R for 12 days shows upregulation of endogenous CTSB, CTSK, RANKL, and Ets2. **(B)** Quantitative analysis of Ets2 expression reveals significant upregulation of Ets2 expression in response to IL-6+IL-6R treatment for 12 days. **(C, D)** CTSK, CTSB, and Ets2 were upregulated specifically in the presence of both IL-6 and IL-6R in human RASFs. **(E)** Human PBMC-derived osteoclast progenitor cells were stimulated with either M-CSF+RANKL or IL-6+IL-6R for 12 days. Western blot analysis of whole cell extracts prepared from these cells showed increased expression of CTSB and Ets2 in response to IL-6 trans-signaling. **(F)** CTSB, CTSK, and Ets2 expression was upregulated in mouse BMDMs in response to M-CSF+RANKL or IL-6+IL-6R. **(G)** Joint homogenates prepared from adjuvant-induced arthritis (AIA) rats show significant upregulation of CTSB, CTSK, RANKL, and Ets2 expression.**p < 0.01 while *p < 0.05.

Human RASFs grown for 12 days under the differentiation with M-CSF/RANKL and/or IL-6/IL-6R were evaluated for osteoclast-specific proteins. Western blot analysis of osteoclast-specific signature proteins confirms upregulation of cathepsin K, cathepsin B, p-STAT-3, RANKL, OPG, and RANKL expression by both M-CSF/RANKL and IL-6/IL-6R treatment (**Figures 2A, B**). Interestingly, while M-CSF/RANKL were able to preferentially activate traditional osteoclast markers such as OSCAR, OPG, and RANKL, IL-6/IL-6R was unique in inducing Ets2 transcription factor, in addition to p-STAT3 activation, to induce cathepsins K and B expression in human RASFs (**Figure 2A**). Furthermore, IL-6 trans-signaling in RASF-induced dose-dependent Ets2 expression by 70%–85% (*p* < 0.01; *n* = 4) and consequently induced cathepsins K and B expression by ~70% and 100%–125%, respectively, compared with untreated RASFs (**Figures 2C, D**; *p* < 0.05 or *p* < 0.01; *n* = 3). To compare these changes to a classical osteoclast model, primary human PBMC-derived macrophages were differentiated into osteoclasts by M-CSF/RANKL or IL-6/IL-6R for 12 days. Western blot analysis showed that IL-6/IL-6R induced the expression of cathepsin B, Ets2, and p-STAT-3 (Ser^705^) that were independent of and comparable with M-CSF/RANKL treatment (**Figure 2E**). Furthermore, BMDM in response to M-CSF/RANKL and IL-6/IL-6R combination showed the marked upregulation of osteoclast-specific cathepsin K, cathepsin B, MITF, and Ets2 expression when compared with M-CSF/RANKL alone treatment (**Figure 2F**). Western blot analysis of joint homogenates prepared from naïve and AIA rats showed a marked increase in the expression of Ets2 along with other bone resorption markers including cathepsin K, cathepsin B, and RANKL (**Figure 2G**), suggesting a potential role of Ets2 in bone resorption in RA.

### Untargeted Proteomics Analysis of the Secretome Reveals RASF Metabolic Reprogramming in Response to Chronic IL-6/IL-6R Exposure

**Figure 3:**
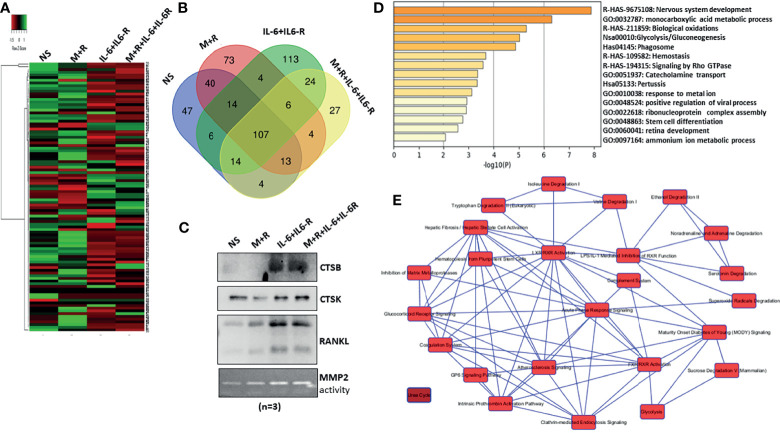
MS-MS analysis of conditioned media identified uniquely expressed protein by IL-6/IL-6R treatment in human RASFs *in vitro*. **(A)** Conditioned media from four RASF donor cells treated with M-CSF/RANKL, IL-6/IL-6R, and M-CSF/RANKL plus IL-6/IL-6R (All) for 12 days were subjected to untargeted proteomics, which revealed 107 common proteins with differential expression. **(B)** Venn diagram shows the number of common and unique secretory proteins identified through untargeted proteomics in RASFs. **(C)** Increased secretion of CTSB, CTSK, and RANKL by transformed RASFs was confirmed through Western blotting of the conditioned media from the treated RASFs. **(D)** Cellular pathway changes brought about by chronic exposure of IL-6+IL-6 R in RASFs were analyzed using Metascape Gene Ontology studies, and **(E)** network analysis using ingenuity pathway analysis which predicted metabolic reprogramming in RASFs.

To characterize the composition of the secretory proteins in response to IL-6/IL-6R stimulation and determine if that reflects the signature of osteoclast-like proteins, the conditioned media obtained from human RASFs untreated or treated with M-CSF/RANKL, IL-6/IL-6R, or the combination of M-CSF/RANKL and IL-6/IL-6R (All) were used for the untargeted proteomic analysis using MS. Initial data analysis identified roughly 107 common secreted proteins from RASFs under various treatments; these are presented with differential spectral count in the heatmap (**Figure 3A**). Further analysis of proteomic data identified 113 proteins unique to the secretome of RASFs treated with IL-6/IL-6R, in contrast to only 73 proteins specific for M-CSF/RANKL stimulation (**Figure 3B**). An additional 27 proteins were identified in both M-CSF/RANKL- and IL-6/IL-6R-stimulated supernatants. Western blot analysis on the concentrated supernatants from these differentiated RASFs confirmed increased production of cathepsin B, cathepsin K, and RANKL in IL-6/IL-6R-activated secretome (**Figure 3C**). Overall, gene signatures of all secretome in response to M-CSF+RANKL, IL-6+IL-6R, and M-CSF+RANKL+IL-6+IL-6R are listed in **Supplementary Table S2**; followed by Gene Ontology studies of M-CSF+RANKL, IL-6+IL-6R, and MCSF+RANKL+IL-6+IL-6R which are also described in **Supplementary Figure S3**. Focusing on the 113 proteins secreted explicitly by IL-6/IL-6R treatment of RASFs, we performed a Gene Ontology study using Metascape and Ingenuity Pathway Analysis (**Figures 3D, E**), revealing the metabolic changes in RASFs. Metascape analysis of the proteomics data identified the metabolic reprogramming of the RASFs as evident from the changes in metabolic pathway-related candidates such as *carboxylic acid metabolism*, *oxidation*, *glycolysis*, *phagocytosis*, *stem cell differentiation*, and *ammonia metabolism* (**Figure 3D**). In addition, the Ingenuity Pathway Analysis predicted the impact on tryptophan, isoleucine, and valine pathways, hematopoiesis from pluripotent stem cells, hepatic fibrosis, retinoid X receptor activation, endocytosis, and the complement system by IL-6/IL-6R treatment (**Figure 3E**). Details of the top 10 molecular networks predicted by IPA in response to IL-6/IL-6R treatment are mentioned in **Supplementary Table S3**.

### IL-6 Trans-Signaling Crosstalk With M-CSF/RANKL and Independent Activation of MAPK Pathway in Human RASFs *In Vitro*

**Figure 4:**
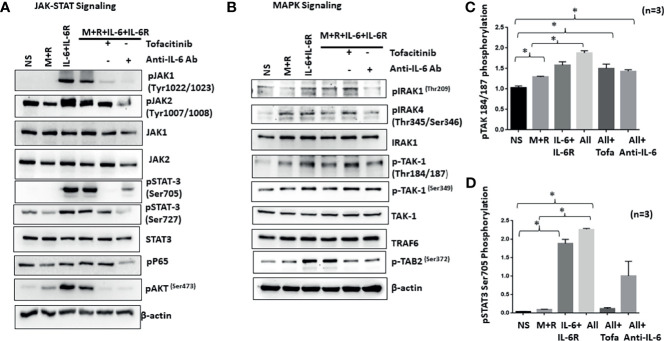
IL-6+IL-6R activates MAPK pathway in addition to canonical JAK-STAT signaling pathway. **(A)** Stimulation of RASFs with M+R (M-CSF+RANK), IL-6+IL-6R, and M+R+IL-6+IL-6R (All) activated the canonical JAK-STAT signaling pathway, evidenced by the increased phosphorylation of JAK1, JAK2, STAT3, and AKT, which was abrogated by tofacitinib and anti-IL-6 antibody treatment. **(B)** M-CSF+RANK, known to engage the MAPK signaling pathway, show cooperativity with IL-6+IL-6R and M+R+IL-6+IL-6R (All)-induced JAK-STAT signaling. Activation of key MAPK proteins by phosphorylation such as TAK1 seems unaffected by tofacitinib or anti-IL-6 antibody pretreatment. **(C, D)** Quantitative analysis of TAK1 phosphorylation at Thr184/187 and phospho-STAT3 Ser 705 shows M+R+IL-6+IL-6R (All) samples are unaffected for phosphorylation of TAK1 in presence of inhibitors. *p < 0.05.

IL-6/IL-6R-initiated trans-signaling activates the JAK/STAT pathway (20), whereas M-CSF/RANKL drives MAPK-dependent signaling for cell survival and differentiation (21). To understand how early signaling events play a role in RASF reprogramming, we stimulated cells with M-CSF/RANKL and/or IL-6/IL-6R for 30 min. Western blot analysis of the cell lysates showed that IL-6/IL-6R selectively induced the activation of p-JAK1 (Tyr^1022/1023^), p-JAK1 (Tyr^1007/1008^), p-STAT3 (Tyr^705^), and p-STAT3 (Ser^727^), without activating NF-κBp65 in RASFs (**Figure 4A**). Interestingly, IL-6/IL-6R induced the expression of p-Akt, a potent cell survival and proliferation marker protein. While M-CSF/RANKL combination was ineffective in activating the JAK/STAT3 pathway itself, the combination partially suppressed IL-6/IL-6R-induced activation of JAK/STAT3 and Akt pathways (**Figure 4A**). Pretreatment of RASFs with tofacitinib or anti-IL-6 antibody showed not only the reduction in JAK/STAT signaling but also suppressed IL-6/IL-6R-induced activation of p-Akt in human RASFs (**Figure 4A**). Interestingly, anti-IL-6 antibody was more effective in reducing the phosphorylation of JAK1 while tofacitinib seems to be more effective on suppressing the phosphorylation of JAK2.

To understand if IL-6 trans-signaling influences M-CSF/RANKL-induced MAPK activation, we analyzed the same samples for proteins upstream of the MAPK pathway (IRAK1/IRAK4/TAK1) in RASF signaling (22). Western blot analysis showed that IL-6/IL-6R markedly induced phosphorylation of p-IRAK4 (Thr^345^/Ser^346^) and p-TAK1 (Thr^184/187^) expression alone or in combination with M-CSF/RANKL in RASFs, which was not effectively inhibited by tofacitinib or anti-IL-6R antibody (**Figure 4B**), suggesting that IL-6/IL-6R and M-CSF/RANKL activation of the MAPK pathways are independent of its JAK-STAT signaling and may remain activated even with IL-6-targeted treatments. Quantitative analysis of TAK1 phosphorylation and STAT3 phosphorylation suggesting TAK1 is unaffected by IL-6 inhibitors (**Figures 4C, D**).

### Ets2 Translocates to the Nucleus in Response to IL-6 Trans-Signaling and Activates the Cathepsin Promoters

**Figure 5:**
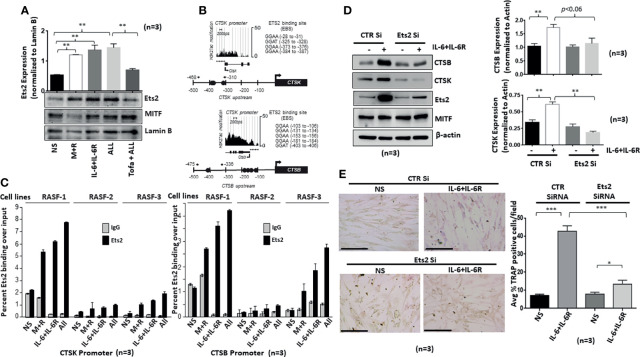
IL-6/IL-6R-induced nuclear translocation of Ets2 and binds to the promoters of *CTSK* and *CTSB* genes to promote osteoclastogenesis in RASFs. **(A)** Ets2 translocates to the nucleus of RASFs within 4 h of IL-6+IL-6R stimulation alone or in combination with M-CSF+RANKL, which was inhibited by tofacitinib. **(B)** Bioinformatic and ChIP-seq analysis on tracks generated by GSE31621 identified the core-binding motif (GGAA/T) for Ets2 and the transcriptionally active regions in the promoters of *CTSK* and *CTSB* genes. **(C)** Chromatin immunoprecipitation using anti-Ets2 antibodies enriched CTSK and CTSB promoters from three independent human RASF donor cells upon stimulation with IL-6+IL-6R and M+R+IL-6+IL-6R (All). **(D)** Ets2 knockdown along with control (CTR), using siRNA, in RASFs treated with IL-6+IL-6R for 12 days resulted in the downregulation of endogenous CTSB and CTSK expression. Bar scale for images acquired is 150 µm. **(E)** Knockdown of Ets2 in RASFs obliterated the IL-6+IL-6R-induced TRAP-positive staining. *p < 0.05, **p < 0.05-p < 0.01, and ***p < 0.001.

We hypothesized that Ets2 activity and binding in osteoclast-specific *CTSK* and *CTSB* gene promoters are mechanistically linked to the differentiation of human RASFs to an osteoclast-like phenotype. To address this hypothesis, we first verified Ets2 nuclear translocation in response to M-CSF/RANKL and/or IL-6/IL-6R combination in the nuclear extract prepared as previously described (23). We observed that M-CSF/RANKL increased the nuclear translocation of Ets2 by twofold (*p* < 0.05; *n* = 3) compared with the untreated samples (**Figure 5A**). Interestingly, IL-6/IL-6R increased Ets2 nuclear content by almost threefold, which was higher than M-CSF/RANKL-stimulated samples and did not increase further in M-CSF/RANKL and IL-6/IL-6R combination (All) treatment (**Figure 5A**; *p* < 0.01; *n* = 3). Ets2 nuclear localization loading control tubulin and lamin B shown in **Supplementary Figure S4**. Aligned to this finding, pretreatment of RASFs with tofacitinib significantly inhibited the combination-triggered nuclear translocation of Ets2 to the level seen in unstimulated samples (**Figure 5A**; *p* < 0.01; *n* = 3), suggesting that JAK/STAT is a pivotal pathway to interfere in this process. We further utilized bioinformatic and ChIP-seq analysis to determine the core-binding motif (GGAA/T) for Ets2 and the transcriptionally active regions in *CTSK* and *CTSB* gene promoters. We observed four Ets2-binding sites in *CTSK* and five in *CTSB* within −500 bps upstream of respective promoters (**Figures 5B, C**). ChIP-seq analysis of previously published GSE31621 datasets further demonstrated that the genomic regions around −500 bps upstream of both the *CTSK* and *CTSB* gene are highly occupied with histone H3K27 acetylation (H3K27ac) modifications (**Figure 5B**). These high levels of the active chromatin marks around Ets2 binding indicate that Ets2 is directly involved in activating *CTSK* and *CTSB* gene expression and promoting differentiation. To further define the involvement of Ets2, we performed ChIP assay in RASFs treated with M-CSF/RANKL, IL-6/IL-6R, or M-CSF/RANKL and IL-6/IL-6R combination for 12 days. The results of the ChIP analysis showed that Ets2-immunoprecipitated DNA showed a significant increase in the occupancy of Ets2 protein in the *CTSK* promoter after treatment with M-CSF/RANKL, IL-6/IL-6R, and their combination when compared with control IgG (**Figure 5C**; *n* = 3). We also found similar enrichment in the *CTSB* promoter. Taken together, our promoter analysis and ChIP assay findings indicate that Ets2 binding is implicated in the osteoclast-like differentiation of RASFs. Furthermore, we performed the repeated knockdown of Ets2 on every 4th day for 12 days in IL-6/IL-6R-treated RASFs, followed by Western blot analysis to observe a reduced expression of IL-6/IL-6R-induced cathepsin B and cathepsin K, which confirms Ets2 transactivation of cathepsin genes (**Figure 5D**; *p* < 0.01, *n* = 3). Our observation of no change in expression of osteoclastic transcription factor MITF suggests that it is not directly involved in IL-6/IL-6R-induced reprogramming of RASFs to osteoclast-like phenotype. Interestingly, the reduced TRAP staining of RASFs on 12 days of IL-6/IL-6R treatment upon Ets2 knockdown further validated its role in the osteoclast-like phenotype of RASFs (**Figure 5E**).

### RASFs Are Predetermined to Be Responsive to IL-6 Trans-Signaling Mediated Differentiation

**Figure 6:**
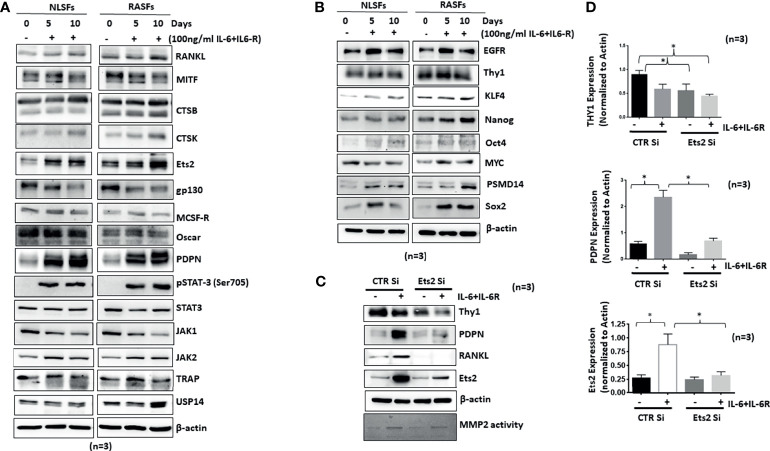
IL-6+IL-6R trans-signaling activates both NLSFs and RASFs to an aggressive and invasive phenotype mediated by Ets2. **(A)** NLSFs and RASFs were differentiated in the presence of IL-6+IL-6R for 5 and 10 days followed by preparation of whole cell extracts that were then probed for osteoclast-specific markers. IL-6 trans-signaling was activated in both NLSFs and RASFs that led to the increased expression of Ets2 which in turn increased the expression of RANKL, CTSK, CTSB, and PDPN. **(B)** IL-6+IL-6R stimulation upregulated the expression of differentiation marker proteins Sox2, Oct4, KLF4, and Nanog higher in RASFs as compared with NLSFs. **(C)** IL-6+IL-6R-induced heterogeneity markers in RASFs, Thy1, and PDPN were downregulated in the absence of Ets2. **(D)** Quantitation of Thy1, PDPN, and Ets2 in 12-day differentiated RASFs confirms more then 80% knockdown of Ets2 as well as significant reduction in PDPN and Thy1 expression. *p < 0.05.

Synovial tissue-derived RASFs possess a unique capability to dedifferentiate and have recently been identified as synovial mesenchymal stem cells (SMCs) (24). To understand how human RASFs respond to IL-6/IL-6R-induced osteoclast-like phenotype and functions compared with nondiseased human SFs (NLSFs), we treated both cell types with IL-6/IL-6R for 5 or 10 days in culture. Western blot analysis of the cell lysates showed selectively higher expression of osteoclast-specific markers (cathepsin K, cathepsin B, RANKL, and Ets2) by RASFs compared with the NLSFs, with no difference in p-STAT3 activation between two groups (**Figure 6A**). To our surprise, we observed a temporal decrease in the endogenous levels of gp130 and JAK1 in both NLSFs and RASFs treated with IL-6/IL-6R. When probed for the stem cell markers, RASF lysates showed markedly higher basal expression level of KLF4, Nanog, Oct4, and Sox-2, along with consistently higher response of these markers to IL-6/IL-6R stimulation compared with NLSFs (**Figure 6B**; *p* < 0.05). This suggests that RASFs are more susceptible than NLSFs to cytokine-driven metabolic reprogramming. Knockdown of Ets2 in RASFs stimulated with IL-6/IL-6R revealed that Ets2 might play a critical role in its invasive phenotype or osteoclast-like phenotype reprogramming by targeting Thy1, podoplanin, and RANKL expression (**Figures 6C, D**). Although IL-6/IL-6R did not induce the expression of Thy1, the knockdown of Ets2 abrogated the expression of Thy1 in untreated and IL-6/IL-6R-treated samples, suggesting its involvement in RASF heterogeneity beyond the regulatory role of IL-6 trans-signaling.

## Discussion

The present study revealed a novel mechanism of IL-6/IL-6R-driven metabolic reprogramming of RASFs to osteoclast-like cells that could contribute to bone destruction in RA. This reprogramming of RASFs to an osteoclastic phenotype is the first study to identify the role of transcription factor Ets2 in IL-6/IL-6R-induced cathepsins K and B and its therapeutic relevance in limiting the bone loss mediated by aggressive and invasive RASFs. We also identified the stem cell markers that were uniquely higher in RASFs when compared with the NLSFs that suggest increased plasticity in activated RASFs to switch their phenotype and function in the synovial microenvironment. These findings shed light on a noncanonical pathway that IL-6/IL-6R exploits to exacerbate bone destruction, highlighting an opportunity to examine the current IL-6 signaling and trans-signaling inhibitors used in the clinics for their efficacy in suppressing the pathological effects of IL-6 mediated through Ets2. Since Ets2 is a transcription factor, it may have some influence on global transcriptomics of RASFs, which may also contribute to the metabolic reprogramming of RASFs as osteoclast-like cells.

IL-6 is a pleiotropic cytokine that supports B-cell proliferation (20) and directly stimulates CRP production from hepatocytes (21). IL-6 drives the chronic inflammation in RA synovial microenvironment and exerts its function through classical and trans-signaling (25, 26). IL-6 trans-signaling is mediated by the binding of IL-6 to the soluble IL-6 receptor (sIL-6R) and signal transduction *via* membrane-bound gp130 to the JAK-STAT pathway. Apart from its role in inflammation, IL-6 signaling is implicated in osteoclastogenesis in RA. The levels of IL-6 and IL-6R in the synovial fluid from RA patients are higher and known to induce osteoclastogenesis in the synovial microenvironment (27). Studies have demonstrated the role of IL-6 trans-signaling in osteoclastogenesis in both osteoclast progenitors and bone marrow-derived cells depending on the presence of IL-6R (28, 29). Both IL-6 and sIL-6R concentrations increase in RA patients’ sera and synovial fluid and correlate with the disease activity (26). We observed IL-6/IL-6R-induced osteoclast-specific TRAP staining, *in vitro* pit formation, cathepsins K and B expression in cultured human RASFs similar to that induced by M-CSF/RANKL, indicating their transformation to osteoclast-like cells. Interestingly, IL-6/IL-6R-driven osteoclastic-like features in RASFs were primarily dependent on Ets2, since its knockdown abolished the production of IL-6/IL-6R-induced bone resorption mediators by RASFs. Previously, Ets2 was shown to be inducible by inflammatory cytokines such as TNF-α and involved in osteoblast reprogramming (30, 31); however, there are no prior publications demonstrating how IL-6 signaling utilizes Ets2 to enhance transcriptional activation of *CTSK* and *CTSB* promoter to cause bone resorption executed by RASFs. These findings provide a rationale for targeting Ets2 transcription factor for reducing bone resorption in RA pathogenesis.

Furthermore, a comprehensive proteomic analysis of the conditioned media from the transformed RASFs revealed 113 proteins secreted exclusively in response to chronic IL-6/IL-6R stimulation, including cathepsins K and B. Further Gene Ontology analysis of this data suggests RASFs undergo a massive metabolic reprogramming that transforms these cells into multipotent stem cell phenotype. It has been previously reported that RASFs are metabolically altered cells with high glycolysis rates which maintain their hyperproliferative rate and aggressive phenotype in the inflamed synovial microenvironment (32–36). Our preliminary findings confirm IL-6/IL-6R trans-signaling engages the metabolic pathways such as glycolysis and the catabolic pathway for sucrose and amino acids (tryptophan, isoleucine, valine) to propel metabolic reprogramming of RASFs. However, we primarily focused our work on examining the functional reprogramming in the influence of chronic IL-6 and predicted pathways obtained from Metascape and IPA analyses of proteomics data may further be subjected to in-depth study for better understanding of the role of IL-6 trans-signaling and Ets2 in metabolic reprogramming of synovial fibroblasts. Oshita et al. reported that mesenchymal stem cells (MSCs) constitutively express osteoprotegerin (OPG) protein (11, 16, 37). Surprisingly, OPG is expressed by RASFs (38) constitutively as well as under stimulation (data not shown), which aligns well with the report by Li et al. (24) classifying RASFs as MSCs. Our data further show that in addition to the activation of JAK-STAT pathway, IL-6/IL-6R simultaneously activated the MAPK signaling pathway in RASFs, as revealed by the increased phosphorylation of IRAK1, IRAK4, and TAK1 proteins. However, the inability of IL-6 inhibitors to target this noncanonical pathway leaves an opportunity for IL-6 to continuously activate the MAPK pathway and potentially contribute to the chronic bone loss in RA. These findings overlap with our observation that Ets2 is central to IL-6/IL-6R-driven bone resorption, partly by activating RASFs to contribute to this process through metabolic reprogramming that coincided with a transition to osteoclast-like phenotype and functions.

Delineating the fundamental role of Ets2 in this pathological process of RASF transformation to osteoclast-like cells could establish Ets2 as a potential therapeutic target in RA. Our ChIP assay using anti-Ets2 antibody revealed the recruitment of Ets2 on the promoters of *CTSK* and *CTSB*, suggesting the direct role of Ets2 in osteoclastic-like changes in RASFs. Interestingly, increased Ets2 expression has been demonstrated in the synovia of some RA patients (39), and the expression of extracellular MMPs has been reported to be driven by Ets2 (40) in invasion and metastasis as well as stem cell self-renewal process (41, 42). However, there is very little published data on the role of Ets2 in RA pathogenesis, and in light of our findings, it warrants further investigation as a potential therapeutic target for the treatment of RA. Given the limitation of tofacitinib and anti-IL-6 Ab to inhibit IL-6-induced trans-signaling through TAK1 suggests that inhibiting Ets2 may have pronounced benefit that is potentially elicited by the suppression of MAPK and JAK/STAT signaling in human RASFs. Our results suggest that RASFs undergo accelerated reprogramming due to IL-6-induced expression of differentiation markers such as Sox2, KLF, OPG, and Nanog, and the hyperinvasive fibroblast marker Thy1, which were inhibited by silencing Ets2. Overall, our findings provide novel evidence for the central role of Ets2 in IL-6 trans-signaling induced cellular reprograming of RASFs to osteoclast-like cells and its contribution to RASF heterogeneity and bone destruction in RA.

Despite being the most ubiquitous stromal cell type in the synovium, RASFs have proven to be difficult to characterize for their molecular functions and repertoire. Recent studies have made progress in identification of different RASF subsets that play unique role in various bone remodeling processes that lead to pannus outgrowth and joint deformities (43–45). Of particular note, RASF subsets that express podoplanin, fibroblast activation protein-α, and CD90/Thy1 are bound to alter extracellular matrix by producing MMP activity, enhance RANKL expression, and stimulate osteoclast differentiation and activation (46–48). Our study provides evidence that in addition to the contribution of RASF to bone destruction, these cells can actively transform functionally into osteoclast-like cells to propagate these processes using Ets2 as a novel mediator. Further *in vivo* studies evaluating the role of Ets2 in functional reprogramming of RASFs to osteoclast-like cells may have therapeutic value in the treatment of RA.

## Data Availability Statement

The original contributions presented in the study are publicly available. This data can be found here: https://www.ebi.ac.uk/pride/ under the accession number PXD028374.

## Ethics Statement

The animal study was reviewed and approved by Washington State University IACUC Committee.

## Author Contributions

AS and SA designed this study and participated in drafting the manuscript. AS, MH, and BM performed experiments and analyzed the data. BW and QH performed ChIP analysis and data interpretation. YS, AB, and BP performed and analyzed the proteomics data. MO performed the quantitative analysis of stained slides. SK performed statistical analysis. All authors contributed to the article and approved the submitted version.

## Funding

Research reported in this publication was supported by National Institute of Arthritis and Musculoskeletal and Skin Diseases of the National Institutes of Health under award number R01 AR072615 (SA) and the Arthritis National Research Foundation under grant award 849781 (AKS).

## Conflict of Interest

The authors declare that the research was conducted in the absence of any commercial or financial relationships that could be construed as a potential conflict of interest.

## Publisher’s Note

All claims expressed in this article are solely those of the authors and do not necessarily represent those of their affiliated organizations, or those of the publisher, the editors and the reviewers. Any product that may be evaluated in this article, or claim that may be made by its manufacturer, is not guaranteed or endorsed by the publisher.
