# Corrigendum: Ets-2 Propagates IL-6 Trans-Signaling Mediated Osteoclast-Like Changes in Human Rheumatoid Arthritis Synovial Fibroblast

**DOI:** 10.3389/fimmu.2021.808756

**Published:** 2021-11-26

**Authors:** Anil K. Singh, Mahamudul Haque, Bhanupriya Madarampalli, Yuanyuan Shi, Benjamin J. Wildman, Abdul Basit, Sadik A. Khuder, Bhagwat Prasad, Quamarul Hassan, Madhu M. Ouseph, Salahuddin Ahmed

**Affiliations:** ^1^ Department of Pharmaceutical Sciences, Washington State University College of Pharmacy, Spokane, WA, United States; ^2^ University of Washington School of Medicine, Seattle, WA, United States; ^3^ Department of Oral and Maxillofacial Surgery, School of Dentistry, University of Alabama at Birmingham, AL, United States; ^4^ Department of Medicine and Public Health, University of Toledo, Toledo, OH, United States; ^5^ Department of Pathology and Laboratory Medicine, Weill Cornell Medical College, New York, NY, United States; ^6^ Division of Rheumatology, University of Washington School of Medicine, Seattle, WA, United States

**Keywords:** osteoclast, interleukin-6, synovial fibroblasts, rheumatoid arthritis, reprogramming

In the published article, there was a mistake in the order of the listed authors. The correct authors list appears below:

“*Anil K. Singh, Mahamudul Haque, Bhanupriya Madarampalli, Yuanyuan Shi, Benjamin J. Wildman, Abdul Basit, Sadik A. Khuder, Bhagwat Prasad, Quamarul Hassan, Madhu M. Ouseph and Salahuddin Ahmed”*.

The authors apologize for this error and state that this does not change the scientific conclusions of the article in any way. The original article has been updated.

## Publisher’s Note

All claims expressed in this article are solely those of the authors and do not necessarily represent those of their affiliated organizations, or those of the publisher, the editors and the reviewers. Any product that may be evaluated in this article, or claim that may be made by its manufacturer, is not guaranteed or endorsed by the publisher.

