# Supplementary material for: Ets-2 Propagates IL-6 Trans-Signaling Mediated Osteoclast-Like Changes in Human Rheumatoid Arthritis Synovial Fibroblast
Source: Front Immunol. 2021 Nov 2;12:746503. doi: 10.3389/fimmu.2021.746503 (PMC8593237; doi:10.3389/fimmu.2021.746503)

SUPPLEMENTARY METHODS

Antibodies and Reagents. Recombinant human IL-6 (Catalog no. 206-IL-200), IL-6R (Catalog no. 227-SR), MC-SF (Catalog no. 216-MC), and IL-6 antibody (MAB2061) were purchased from R&D Systems (Minneapolis, MN). Recombinant human RANKL (Catalo no. 310-01) was purchased from Peprotech (Cranbury, NJ). Rabbit anti-Cathepsin B (Catalog no.), p-TAK1 $Thr^{184/187}$(Catalog no.), MITF (Catalog no.), p-Stat-3 (Tyr^{705}) (Catalog no. 9145), p-Stat-3 (Ser^{727}) (Catalog no. 9134), TRAF6 (Catalog no.8028) MC-SF receptor (Catalog no. 3152), MyD88 (Catalog no. 4283) p-Jak1 ($Tyr^{1034/1035}$) (Catalog no0411. 3331) p-Jak2 ($Tyr^{1007/1008}$) (Catalog no. 3776), p-Akt (Ser^{473}) (Catalog no. 4060) p-NF-κBp65 (Ser^{536}) (Catalog no. 3033) anti-rabbit, and anti-mouse horseradish peroxidase-linked secondary antibodies were purchased from Cell Signaling Technology (Danvers, MA). Anti-OPG (Catalog no. sc-390518), RANKL (Catalog no. sc-377079), Ets2 (Catalog no. sc-365666), Cathepsin K (Catalog no. sc-48353), TAK1 (Catalog no. sc-166562), TRAP (Catalog no. sc-376875), PU.1 (Catalog no. sc-390405), Stat3 (Catalog no. sc-8019), KLF (Catalog no. sc-166238), Oct4 (Catalog no. sc-365509), Podoplanin (Catalog no. sc-376695), Nanog (Catalog no. sc-134218), UCHL5 (Catalog no. sc-98673), Sox2 (Catalog no. sc-365964) β-actin (Catalog no. sc-47778), USP14 (Catalog no. sc-398009) and anti-gp130 antibody (Catalog no. sc-376280) were purchased from Santa Cruz Biotech (Santa Cruz, CA). Rabbit monoclonal p-IRAK1 (Thr^{209}) (Catalog no. SAB4504246) was purchased from Sigma (St. Louis, MO). Anti-OSCAR antibody (Cat# MBS2090644) was purchased from MyBioSource (San Diego, CA).

Flow Cytometry. Flow cytometry was performed to verify RASF cell lines were free from monocyte contamination. Briefly, 3 independent RASFs from different human subject along with human PBMC cells were washed with PBS-based FACS buffer containing 2% fetal bovine serum and 1mM EDTA. All cells were Fc blocked using Biolegend FcX (422302) per the manufacturer's instructions. Blocked cells were

then washed with FACS buffer and stained in primary anti-CD14 antibody (325602) for 30 minutes. Stained cells were washed again followed by secondary staining with Invitrogen AlexaFluor 488 (A-21202) for 30 minutes. Secondary stained cells were washed again followed by Live/Dead staining with 7AAD (420404) for 10 minutes, washed a final time and flow cytometry was performed using a Beckman-Coulter Gallios flow cytometer. Analysis was performed using Kaluza Analysis software version 2.1.2.

Small interfering RNA. Small interfering RNA (siRNA) sequences for Ets2 were purchased from Millipore Sigma (eMISSION predesigned siRNA sequences SASI_Hs01_00130873 and SASI_Hs01_00130872. RASFs were transfected with 100 pmol of scramble (SIC001) and Ets2 siRNA with Lipofectamine 2000 (Thermo Fisher Scientific, Waltham, MA, USA) as per manufacturer instruction. Cells were grown in complete RPMI medium with or without 100ng/ml IL6 +100ng/ml IL6R for 12 days. To maintain continuous knockdown while differentiation of RASFs every transfection step was repeated on consecutive 3 times on day 3, 6 and 9. On day 11 of transfection, RASFs were serum starved overnight along with 100ng/ml IL6 +100ng/ml IL6R simulation for overnight.

Gelatin Zymography: Cell supernatant from MCSF+RANKL, IL6+IL6R and MCSF+RANKL+ IL6+IL6R from 12 day differentiated fibroblast were subjected to gelatin zymography using Novex gel (cat: Zy00100BOX) from Invitrogen as per manufacturer instruction. MMP2 bands were photographed using the Bio-Rad XRS system.

Pit formation assay analysis: For analysis of the pit in the calcium phosphate coated plate used image-J Fiji software which is freely available from the NIH. briefly we have a pit (which is stained with toluidine blue for the visibility) we draw a random boundary based on the pit area for bigger or smaller or any size which we can see blue color we drew hands free lines. We added all the area (size of area in field) from all the hand drawn pit outlines and divided it from overall size of the field (that gives us total blue area in overall field which is also proportional to cumulative of pit area and multiplied by 100 to get percentage value. We repeated this at least in an N-9 independent field to get % of pit relative to each other.

Western Immunoblotting. To study the effect on RASF differentiation and phenotypic switch, the whole-cell extracts were prepared using RIPA buffer (50 mM Tris pH 7.6, 150 mM NaCl, 1% Triton X100, 1mM EDTA, 0.5% sodium deoxycholate, 0.1% SDS) containing Complete mini-protease inhibitor and Phospho-Stop tablets (Roche, Indianapolis, IN). The amount of protein was measured using the Bio-Rad DC method (Bio-Rad, CA). Equal amounts of protein (25 µg) were loaded and separated by SDS-polyacrylamide gel electrophoresis and transferred onto PVDF membranes (EMD Millipore). Blots were probed using respective primary blots followed by strip and re-probed for other primary antibodies or β-actin as an endogenous control. Densitometric analysis of the relative expression of each protein was done as described earlier (22). For determining soluble proteins, the Western blotting was performed on the conditioned media collected from the treated samples at different time points and concentrated 500 µL using a 3-kDa cutoff Amicon filters (Millipore).

Chromatin immunoprecipitation assay (ChIP). Human RASFs crosslinking was performed by adding formaldehyde to a final concentration of 1% at room temperature for 10 minutes, and the reaction stopped by the addition of 125 mM glycine. Cells were washed with ice-cold phosphate-buffered saline containing 0.1 mM PMSF. Cell pellet was resuspended in 1 mL of ChIP sonication buffer (50 mM Tris-HCl, pH 8, 1% Triton X-100, 0.5% Deoxycholate, 150 mM NaCl, 1 mM EDTA, 0.3% SDS, Complete mini and Phos STOP protease inhibitors) and placed on ice for 30 minutes. DNA was sheared by sonication, and the cell debris was pelleted by centrifugation at 15,000 *g* for 15 minutes. Forty µl of clear lysate was taken out to check quality controls of sonication. Sonication quality was checked on an agarose gel, and leftovers were stored as input for further use. The supernatant was collected and pre-cleared with 50 µl of Protein G Sepharose (Roche, IN) equilibrated with Baker's Yeast tRNA (Sigma) for 60 minutes at 4 ºC. The pre-cleared

whole-cell extract was equally divided into three parts and three times diluted in Chip dilution buffer (Chip Sonication buffer without SDS). The diluted samples were incubated with 5 µg of Ets2 or IgG antibody at 4° C overnight. 10% of the volume was frozen and kept aside as input. The immune complex was precipitated by incubation of the samples with 50µl Protein G Sepharose equilibrated with tRNA at 4 °C for 4 hours. The samples were centrifuged at 500 g at 4 °C for 1 minute, and the supernatant was discarded. The Protein G Sepharose beads were washed with cold ChIP dilution buffer containing protease inhibitors followed by a wash with low salt wash buffer (1% TritonX-100, 0.1% sodium deoxycholate, 50 mM Tris pH 8.0, 150 mM NaCl and 5 mM EDTA), high salt wash buffer (1% Triton X-100, 0.1% sodium deoxycholate, 50 mM Tris pH 8.1, 500 mM NaCl and 5 mM EDTA), LiCl wash buffer (0.25 M LiCl, 0.5% Triton X100, 0.5% sodium deoxycholate, 1 mM EDTA and 10 mM Tris pH 8.1) and finally with 1X TE pH 8.

The immune complex was eluted with 1% SDS, 0.1 M $NaHCO_3$. Crosslinking was reversed by incubating at 65°C overnight with 2µg RNAse A followed by 2 hours of 5µl proteinase K (20 mg/mL) treatment at 65°C. After Proteinase K digestion, immunoprecipitated DNA was subjected to ethanol precipitation overnight at -20°C. Extracted DNA was further purified using a PCR purification column (Qiagen). PCR was performed using corresponding distal, mid, and proximal sequences to Cathepsin K and Cathepsin B gene promoters. IDT and the sequences of the synthesized primers are listed in Table S1. Additionally preliminary bioinformatics analysis was performed on CTSK and CTSB promoter by generating track hubs for the chip datasets which are available at http://www.ag-rehli.de. GSE31621 from NCBI GEO data were used for temple to generate track.

Untargeted proteomics of RASFs. Two µl of the sample (~100 ng/µl protein) was injected into a 100 µm ID Integrafrit trap (New Objective) packed with Reprosil-Pur C18-AQ 120 Å 5 µm

material to a bed length of 2.5 cm at a flow rate of 2 µL/min. After loading and desalting for 10 min with 0.1% formic acid plus 2% acetonitrile, the trap was brought in-line with a pulled fused-silica capillary tip (75-µm i.d.) packed with 35 cm of Reprosil-Pur C18-AQ 120 Å 5 µm. Peptides were separated using a linear gradient from 5-30% solvent B (0.1 % formic acid in acetonitrile) in 90 min at a flow rate of 300 L/min. The column temperature was maintained at a constant 50 °C during all experiments. Peptides were detected using the data-dependent acquisition (DDA) method. Survey scans of peptide precursors were performed in the Orbitrap mass analyzer from 375 to 1575 m/z at 120K resolution (at 200 m/z) with a 7 x e5 ion count target a maximum injection time of 50 ms. The instrument was set to run in full speed mode with a 3-sec cycle for the survey and the MS/MS scans. Higher-energy collisional dissociation (HCD) fragmentation was applied with a normalized collision energy of 30%. Resulting fragments were detected in the Orbitrap mass analyzer at 30 K resolution (at 200 m/z) with a 1xe4 ion count target and a maximum injection time of 100 ms. The dynamic exclusion was set to 30 s with a 20 ppm mass tolerance around the precursor and its isotopes.

For untargeted proteomics profiling, tandem mass spectra were analyzed using a variety of bioinformatics tools. All the raw data were converted to mzXML files for database searching and further analyzed by Trans-Proteomic Pipeline using PeptideProphet (Comet), iProphet, Protein Prophet, and StPeter. A target protein database was created by using the UniProt human database. All searches were configured to use a variable modification of methionine oxidation and carbamidomethylation of cysteine as a fixed modification. The results were filtered to a less than 1% protein false discovery rate (FDR) with the requirement for a minimum of one signature peptide per reported protein. The parameters used include: i) minimum peptide length of 6 amino acid, ii) maximum FDR of 1% and protein identification probability more than 0.85, iii) minimum

unique peptides per protein of 1, and modifications to cysteine or methionine were not considered distinct from the unmodified peptides and, iv) tryptic digestion with maximum of two miscleavages allowed. STRING was used for pathway analysis.

Animal experiments. Five wild type control C57BL/6 female mice (8-week-old) were provided by Washington State University animal care facility at Spokane, WA. Briefly, animals were euthanized by CO_2 asphyxiation followed by which hind limbs were harvested and stored in PBS solution. Before pursuing cell isolation from tibia and femur bone were soaked with 70% ethanol for 2-3 minutes followed by cutting of ends using a surgical grade scissors. Bone marrow was flushed out using a 25-gauge needle using PBS in a sterile tube containing penicillium-streptomycin containing RPMI without serum. Collected cells were dispersed using 5 ml pipettes followed by ficoll density centrifugation at 400 g rpm in a swinging bucket rotor. Further buffy phases containing all mononuclear suspension cells were collected using 10 ml pipette. Cells were further washed 2 to 3 times in plain RPMI-1640 and counted before proceeding to various treatments as mentioned in the method section for *Cell culture and stimulation.*

To determine protein expression in rat inflammatory arthritis, joint homogenates were considered from our previously published study [DOI: 10.1002/art.39447] for only naïve and adjuvant-induced arthritis (AIA) joint homogenates to be used for determining the expression levels of CTSB, CTSK, RANKL, Ets2 and β-actin.

SUPPLEMENTARY TABLES

Table 1: Sequence of primer pair for CTSB, CTSK and Ets2 respective to Transcription start site

**CTSB Primers -242 to -359**	**CTSB Primers +14 to +154**
**F1:** TCCTCAGTTCCTCGGGTATCC	**F2:** TTACTAGAGACGGGGTTTCACC
**R1:** GTACTCCCGCCTGGTTAGTAAC	**R2:** GGCGACAGTAAGAAGGCAGTAG
**CTSK Primers +39 to -99**	**CTSK Primers -232 to -333**
**F1:** AGAAACACTGCAAATCCACTGC	**F2:** TCCTAACAGGAAAGGGGTAGGA
**R1:** TTTATTCCATCGGGATTGCGGA	**R2:** AAAAGGAAGGACACAGCCCTAG
**ETS2 Primers -24 to -220**	**ETS2 Primers -397 to -240**
**F1:** CTCCGAGAGTGACGATGATGTG	**F2:** CATGGACTCAAGCACCTTCTGA
**R1:** CGCTTCCTGTAGAAACCGGG	**R2:** CACAGGTGGGAACTGAACCTTA

Table S2: Secretome of RASFs in response to NS, MCSF+RANKL (M/R), IL-6/IL-6R and MCSF+RANKL+IL6+IL6R (M/R/IL-6/IL-6R)

NS	M/R	IL-6/IL-6R	M/R/IL-6/IL-6R
A0A024R6I7_HUMAN	A0A024R6I7_HUMAN	3HAO_HUMAN	A0A024R6I7_HUMAN
A0A087WTA8_HUMAN	A0A087WSY5_HUMAN	A0A087WTA8_HUMAN	A0A087WTA8_HUMAN
A0A087WWT3_HUMAN	A0A087WTA8_HUMAN	A0A087WWT3_HUMAN	A0A087WWT3_HUMAN
A0A087WXH5_HUMAN	A0A087WW88_HUMAN	A0A087WYF1_HUMAN	A0A087WZP6_HUMAN
A0A087WYC1_HUMAN	A0A087WWQ4_HUMAN	A0A087WYV8_HUMAN	A0A087X0S5_HUMAN
A0A087WYF1_HUMAN	A0A087WWT3_HUMAN	A0A087X054_HUMAN	A0A0A0MQX7_HUMAN
A0A087X0S5_HUMAN	A0A087WYX9_HUMAN	A0A087X0S5_HUMAN	A0A0A0MS08_HUMAN
A0A087X1S9_HUMAN	A0A087X0S5_HUMAN	A0A0A0MS08_HUMAN	A0A0A0MT01_HUMAN
A0A087X1T7_HUMAN	A0A087X1T7_HUMAN	A0A0A0MT01_HUMAN	A0A0A0MTC7_HUMAN
A0A0A0MQS9_HUMAN	A0A0A0MQS9_HUMAN	A0A0A0MTC7_HUMAN	A0A0B4J231_HUMAN
A0A0A0MQX7_HUMAN	A0A0A0MQX7_HUMAN	A0A0B4J1R6_HUMAN	A0A0G2JMB2_HUMAN
A0A0A0MS08_HUMAN	A0A0A0MS08_HUMAN	A0A0C4DFS0_HUMAN	A0A0G2JPA8_HUMAN
A0A0A0MT01_HUMAN	A0A0A0MT01_HUMAN	A0A0C4DG90_HUMAN	A0A0U1RQF0_HUMAN
A0A0B4J231_HUMAN	A0A0B4J231_HUMAN	A0A0D9SFB5_HUMAN	A0A0U1RQV3_HUMAN
A0A0G2JMB2_HUMAN	A0A0G2JMB2_HUMAN	A0A0G2JMB2_HUMAN	A0A140TA49_HUMAN
A0A0G2JPA8_HUMAN	A0A0G2JPA8_HUMAN	A0A0G2JPA8_HUMAN	A0A1B0GUU9_HUMAN
A0A0U1RQK7_HUMAN	A0A0U1RQK7_HUMAN	A0A0G2JS28_HUMAN	A0A286YES1_HUMAN
A0A0U1RQV3_HUMAN	A0A0U1RQV3_HUMAN	A0A0U1RQF0_HUMAN	A0A286YEY1_HUMAN

NS	M/R	IL-6/IL-6R	M/R/IL-6/IL-6R
A0A140TA49_HUMAN	A0A140TA49_HUMAN	A0A140TA49_HUMAN	A0A286YEY4_HUMAN
A0A1W2PS52_HUMAN	A0A1B0GTX1_HUMAN	A0A1B0GUU9_HUMAN	A0A286YFJ8_HUMAN
A0A286YES1_HUMAN	A0A1B0GWE8_HUMAN	A0A1B0GW33_HUMAN	A0A2Q2TTZ9_HUMAN
A0A286YEY1_HUMAN	A0A1X7SC65_HUMAN	A0A1B0GWE8_HUMAN	A0A2R8Y7C0_HUMAN
A0A286YEY4_HUMAN	A0A286YES1_HUMAN	A0A286YES1_HUMAN	A0A3B3IS80_HUMAN
A0A286YFJ8_HUMAN	A0A286YEY1_HUMAN	A0A286YEY1_HUMAN	A0A3B3IT64_HUMAN
A0A2Q2TTZ9_HUMAN	A0A286YEY4_HUMAN	A0A286YEY4_HUMAN	A0A3B3ITK0_HUMAN
A0A2R8Y7C0_HUMAN	A0A286YFJ8_HUMAN	A0A286YFJ8_HUMAN	A1AG1_HUMAN
A0A2R8YGX3_HUMAN	A0A2R8Y7C0_HUMAN	A0A2Q2TTZ9_HUMAN	A1AG2_HUMAN
A0A3B3IS80_HUMAN	A0A2R8YCL0_HUMAN	A0A2R8Y6G6_HUMAN	A1AT_HUMAN
A0A3B3ITK0_HUMAN	A0A2R8YGD1_HUMAN	A0A2R8Y793_HUMAN	A1BG_HUMAN
A1AG1_HUMAN	A0A3B3ISX9_HUMAN	A0A2R8YGD1_HUMAN	A2GL_HUMAN
A1AG2_HUMAN	A0A3B3ITK0_HUMAN	A0A3B3IS80_HUMAN	A2MG_HUMAN
A1AT_HUMAN	A0A3B3IU93_HUMAN	A0A3B3ITK0_HUMAN	A6NIW5_HUMAN
A1BG_HUMAN	A1AG1_HUMAN	A1AG1_HUMAN	A8MUN2_HUMAN
A2GL_HUMAN	A1AG2_HUMAN	A1AG2_HUMAN	ADH1A_HUMAN
A2MG_HUMAN	A1AT_HUMAN	A1AT_HUMAN	AFAM_HUMAN
A6XND1_HUMAN	A1BG_HUMAN	A1BG_HUMAN	ALBU_HUMAN
AACT_HUMAN	A2GL_HUMAN	A2GL_HUMAN	AMBP_HUMAN
ACTG_HUMAN	A2MG_HUMAN	A2MG_HUMAN	ANGT_HUMAN
ACTN4_HUMAN	A6XND1_HUMAN	A6NIW5_HUMAN	ANT3_HUMAN
AFAM_HUMAN	A8MUN2_HUMAN	A8MUN2_HUMAN	APOH_HUMAN
ALBU_HUMAN	A8MXR1_HUMAN	AACT_HUMAN	ATRN_HUMAN
AMBP_HUMAN	ACTG_HUMAN	ACO13_HUMAN	ATS1_HUMAN
AMPN_HUMAN	ACTN4_HUMAN	ADAM8_HUMAN	B0YJC5_HUMAN
ANC2_HUMAN	AFAM_HUMAN	ADH1A_HUMAN	B1AHL2_HUMAN
ANGT_HUMAN	ALBU_HUMAN	ADHX_HUMAN	B2MG_HUMAN
ANT3_HUMAN	AMBP_HUMAN	AFAM_HUMAN	B4DPQ0_HUMAN
ANXA5_HUMAN	AMPN_HUMAN	AL1A1_HUMAN	B4E1Z4_HUMAN
APOB_HUMAN	ANGT_HUMAN	AL1L1_HUMAN	B5MCZ3_HUMAN
APOH_HUMAN	ANT3_HUMAN	AL4A1_HUMAN	B7WNR0_HUMAN
ATL3_HUMAN	ANXA1_HUMAN	ALAT1_HUMAN	B7WPK3_HUMAN
ATRN_HUMAN	ANXA5_HUMAN	ALBU_HUMAN	BGH3_HUMAN
ATS1_HUMAN	APOA1_HUMAN	ALDH2_HUMAN	C1S_HUMAN
B0YJC4_HUMAN	APOH_HUMAN	AMBP_HUMAN	C9JIZ6_HUMAN
B1AHL2_HUMAN	AT2B4_HUMAN	ANGT_HUMAN	C9JXI5_HUMAN
B1AP58_HUMAN	ATL3_HUMAN	ANT3_HUMAN	C9JXX4_HUMAN
B2MG_HUMAN	ATRN_HUMAN	ANXA5_HUMAN	CAD13_HUMAN
B4DPQ0_HUMAN	B0YJC4_HUMAN	AOFB_HUMAN	CATB_HUMAN
B4E1Z4_HUMAN	B1AHL2_HUMAN	AOXA_HUMAN	CATL1_HUMAN

NS	M/R	IL-6/IL-6R	M/R/IL-6/IL-6R
B7ZKJ8_HUMAN	B1AP58_HUMAN	APOH_HUMAN	CD109_HUMAN
BASP1_HUMAN	B2MG_HUMAN	ARGI1_HUMAN	CFAH_HUMAN
BGH3_HUMAN	B4DNG0_HUMAN	ARK73_HUMAN	CH3L1_HUMAN
BIP_HUMAN	B4DPQ0_HUMAN	ASSY_HUMAN	CH3L2_HUMAN
BRWD1_HUMAN	B4E1Z4_HUMAN	ATL3_HUMAN	CH60_HUMAN
C1S_HUMAN	B7WNR0_HUMAN	ATRN_HUMAN	CLUS_HUMAN
C9J879_HUMAN	BASP1_HUMAN	ATS1_HUMAN	CO1A1_HUMAN
C9JBF1_HUMAN	BGH3_HUMAN	B0YJC4_HUMAN	CO3_HUMAN
C9JFR7_HUMAN	BTD_HUMAN	B1AH49_HUMAN	CO3A1_HUMAN
C9JG63_HUMAN	C1S_HUMAN	B1AHL2_HUMAN	CO5A1_HUMAN
C9JIZ6_HUMAN	C9JIZ6_HUMAN	B2MG_HUMAN	CO6A2_HUMAN
C9JQS6_HUMAN	C9JYX9_HUMAN	B4DPQ0_HUMAN	CSF1-3_HUMAN
C9JXI5_HUMAN	CAD13_HUMAN	B4E1Z4_HUMAN	CYTC_HUMAN
C9JXX4_HUMAN	CALU_HUMAN	B5MCZ3_HUMAN	D6RF35_HUMAN
CAD13_HUMAN	CATA_HUMAN	B7WNR0_HUMAN	D6RGG3_HUMAN
CALU_HUMAN	CATB_HUMAN	B7WPK3_HUMAN	DNER_HUMAN
CATB_HUMAN	CATK_HUMAN	BIP_HUMAN	E5RJH0_HUMAN
CATL1_HUMAN	CATL1_HUMAN	C1RL_HUMAN	E7ER44_HUMAN
CATZ_HUMAN	CATZ_HUMAN	C1S_HUMAN	E7ET33_HUMAN
CD109_HUMAN	CBPQ_HUMAN	C9J2C0_HUMAN	E7EUF1_HUMAN
CH3L1_HUMAN	CC180_HUMAN	C9JDL1_HUMAN	E9PC41_HUMAN
CLUS_HUMAN	CD109_HUMAN	C9JH92_HUMAN	E9PF17_HUMAN
CO1A1_HUMAN	CH3L1_HUMAN	C9JIZ6_HUMAN	E9PGN7_HUMAN
CO3_HUMAN	CLUS_HUMAN	C9JXI5_HUMAN	E9PHK0_HUMAN
CO3A1_HUMAN	CN183_HUMAN	C9JXX4_HUMAN	ECM1_HUMAN
CO5_HUMAN	CO1A1_HUMAN	CATA_HUMAN	ERN1_HUMAN
CO6A2_HUMAN	CO3_HUMAN	CATB_HUMAN	F5GXS0_HUMAN
CSPG2_HUMAN	CO3A1_HUMAN	CATL1_HUMAN	F5GYU2_HUMAN
CSTN1_HUMAN	CO5A1_HUMAN	CH3L1_HUMAN	F5H4R2_HUMAN
CTGF_HUMAN	CO6A2_HUMAN	CH3L2_HUMAN	F8VRR2_HUMAN
CYR61_HUMAN	CSF1-3_HUMAN	CH60_HUMAN	F8W696_HUMAN
CYTC_HUMAN	CSPG2_HUMAN	CLUS_HUMAN	F8W914_HUMAN
D6RAR4_HUMAN	CSTN1_HUMAN	CO1A1_HUMAN	FBN1_HUMAN
D6RF35_HUMAN	CTGF_HUMAN	CO3_HUMAN	FETUA_HUMAN
D6RF94_HUMAN	CYTC_HUMAN	CO3A1_HUMAN	FETUB_HUMAN
D6RGG3_HUMAN	D6RB01_HUMAN	CO5A1_HUMAN	FINC_HUMAN
DAG1_HUMAN	D6RF35_HUMAN	CO6A2_HUMAN	FSTL1_HUMAN
DC8L2_HUMAN	D6RF94_HUMAN	CPSM_HUMAN	G3V3A0_HUMAN
E7ENL6_HUMAN	D6RGG3_HUMAN	CXCL7_HUMAN	G3XAI2_HUMAN
E7ER44_HUMAN	DAG1_HUMAN	CYTC_HUMAN	G3XAM2_HUMAN

NS	M/R	IL-6/IL-6R	M/R/IL-6/IL-6R
E7ET33_HUMAN	DC8L2_HUMAN	D6RAM7_HUMAN	G3XAP6_HUMAN
E7EUF1_HUMAN	E7ENL6_HUMAN	D6RD47_HUMAN	G5EA25_HUMAN
E9PG08_HUMAN	E7ER44_HUMAN	D6RF35_HUMAN	GDN_HUMAN
E9PHK0_HUMAN	E7ET33_HUMAN	D6RF94_HUMAN	H0Y3T6_HUMAN
E9PIT3_HUMAN	E7EUF1_HUMAN	D6RGG3_HUMAN	H0Y875_HUMAN
E9PKP4_HUMAN	E9PD24_HUMAN	DC8L2_HUMAN	H0Y9C9_HUMAN
E9PR70_HUMAN	E9PFZ2_HUMAN	DHE3_HUMAN	H0YAC1_HUMAN
ECM1_HUMAN	E9PG08_HUMAN	E5RFV2_HUMAN	H0YBX3_HUMAN
ERN1_HUMAN	E9PHK0_HUMAN	E5RJH0_HUMAN	H0YDL9_HUMAN
F5H4R2_HUMAN	E9PHW4_HUMAN	E7ENL6_HUMAN	H0YDX6_HUMAN
F8VRR2_HUMAN	E9PIT3_HUMAN	E7ER44_HUMAN	H0YHM6_HUMAN
F8VYK9_HUMAN	E9PNW4_HUMAN	E7ET33_HUMAN	H0YL56_HUMAN
F8W696_HUMAN	E9PPB3_HUMAN	E7EUC7_HUMAN	H0YMF1_HUMAN
F8W809_HUMAN	E9PRS3_HUMAN	E7EUF1_HUMAN	H0YN42_HUMAN
F8W914_HUMAN	ECM1_HUMAN	E7EUT5_HUMAN	H3BS10_HUMAN
F8WD96_HUMAN	F5GX75_HUMAN	E9PC41_HUMAN	H7C0L5_HUMAN
F8WF14_HUMAN	F5GXJ9_HUMAN	E9PF17_HUMAN	H7C1J8_HUMAN
FABPL_HUMAN	F5H4R2_HUMAN	E9PF18_HUMAN	H7C4N8_HUMAN
FBN1_HUMAN	F5H4V9_HUMAN	E9PG08_HUMAN	H7C5R1_HUMAN
FETUA_HUMAN	F8VRR2_HUMAN	E9PHK0_HUMAN	HBB_HUMAN
FINC_HUMAN	F8W914_HUMAN	E9PIT3_HUMAN	HEMO_HUMAN
FRIL_HUMAN	F8WAD8_HUMAN	E9PKP4_HUMAN	HMCN1_HUMAN
FSTL1_HUMAN	F8WBI9_HUMAN	ECHM_HUMAN	HPT_HUMAN
G3XAI2_HUMAN	F8WE65_HUMAN	ECM1_HUMAN	I3L4N8_HUMAN
G3XAM2_HUMAN	FAM3C_HUMAN	EF2_HUMAN	IBP4_HUMAN
G3XAP6_HUMAN	FBLN1_HUMAN	ERN1_HUMAN	IBP6_HUMAN
G5E9R7_HUMAN	FBLN2_HUMAN	EST1_HUMAN	IBP7_HUMAN
GAS6_HUMAN	FBN1_HUMAN	F2Z349_HUMAN	IGKC_HUMAN
GDN_HUMAN	FETUA_HUMAN	F2Z3J9_HUMAN	IGLC3_HUMAN
GGH_HUMAN	FINC_HUMAN	F5GXS0_HUMAN	IL6RA_HUMAN
H0Y2X5_HUMAN	FRAS1_HUMAN	F5GYU2_HUMAN	ISLR_HUMAN
H0Y3T6_HUMAN	FSTL1_HUMAN	F5H2F4_HUMAN	J3KMX3_HUMAN
H0YAC1_HUMAN	G3V595_HUMAN	F5H4R2_HUMAN	J3KMY5_HUMAN
H0YDL9_HUMAN	G3V5P6_HUMAN	F6SYF8_HUMAN	J3KQ18_HUMAN
H0YDW7_HUMAN	G3XAI2_HUMAN	F8VRR2_HUMAN	J3QSU6_HUMAN
H0YFL7_HUMAN	G3XAP6_HUMAN	F8VZY9_HUMAN	K1C10_HUMAN
H0YHM6_HUMAN	GAS6_HUMAN	F8W1U3_HUMAN	K1C9_HUMAN
H0YN42_HUMAN	GDN_HUMAN	F8W696_HUMAN	K22E_HUMAN
H3BS10_HUMAN	H0Y3T6_HUMAN	F8W8W4_HUMAN	K2C1_HUMAN
H3BT58_HUMAN	H0YAC1_HUMAN	F8W914_HUMAN	K7EKZ5_HUMAN

NS	M/R	IL-6/IL-6R	M/R/IL-6/IL-6R
H7BYH4_HUMAN	H0YCE2_HUMAN	F8W978_HUMAN	K7ENL6_HUMAN
H7C543_HUMAN	H0YD18_HUMAN	F8WE65_HUMAN	K7ERG9_HUMAN
H7C5R1_HUMAN	H0YDW7_HUMAN	FABPL_HUMAN	KIF4A_HUMAN
HBB_HUMAN	H0YFL7_HUMAN	FBLN3_HUMAN	KNG1-3_HUMAN
HEMO_HUMAN	H0YH80_HUMAN	FBN1_HUMAN	LAMC1_HUMAN
HEP2_HUMAN	H0YMJ5_HUMAN	FETUA_HUMAN	LG3BP_HUMAN
HMCN1_HUMAN	H0YN42_HUMAN	FINC_HUMAN	LRP1B_HUMAN
HMCN2_HUMAN	H3BR81_HUMAN	FSTL1_HUMAN	LRP8_HUMAN
HPT_HUMAN	H3BS10_HUMAN	FUMH_HUMAN	LUM_HUMAN
HPTR_HUMAN	H7BYH4_HUMAN	G3V1A4_HUMAN	MEG10_HUMAN
HSP76_HUMAN	H7C0L5_HUMAN	G3XAI2_HUMAN	MFAP1_HUMAN
IBP4_HUMAN	H7C0V9_HUMAN	G3XAM2_HUMAN	MMP2_HUMAN
IBP7_HUMAN	H7C1J5_HUMAN	G3XAP6_HUMAN	NOTC2_HUMAN
IC1_HUMAN	H7C5Q1_HUMAN	G5E9S2_HUMAN	NTF3_HUMAN
IDS_HUMAN	HBB_HUMAN	GDN-3_HUMAN	NUCB1_HUMAN
IGKC_HUMAN	HBD_HUMAN	GPDA_HUMAN	PAI1_HUMAN
IGLC3_HUMAN	HEMO_HUMAN	GSTA3_HUMAN	PCOC1_HUMAN
ISLR_HUMAN	HEP2_HUMAN	H0Y3T6_HUMAN	PEDF_HUMAN
J3KMX3_HUMAN	HMCN1_HUMAN	H0Y532_HUMAN	PGRP2_HUMAN
J3KMY5_HUMAN	HPT_HUMAN	H0Y6I0_HUMAN	PGS1_HUMAN
J3KQ18_HUMAN	HTRA1_HUMAN	H0Y875_HUMAN	PGS2_HUMAN
J3KQJ1_HUMAN	IBP4_HUMAN	H0Y9B1_HUMAN	PI16_HUMAN
J3KQJ9_HUMAN	IBP5_HUMAN	H0YAC1_HUMAN	PPIB_HUMAN
J3QSU6_HUMAN	IBP6_HUMAN	H0YBX3_HUMAN	PRD10-6_HUMAN
K1C10_HUMAN	IBP7_HUMAN	H0YCY6_HUMAN	PSB9_HUMAN
K1C9_HUMAN	IC1_HUMAN	H0YDX6_HUMAN	PTX3_HUMAN
K22E_HUMAN	IGKC_HUMAN	H0YI09_HUMAN	PZP_HUMAN
K2C1_HUMAN	IGLC3_HUMAN	H0YI37_HUMAN	Q4VY20_HUMAN
K7EJB9_HUMAN	ISLR_HUMAN	H0YLA4_HUMAN	Q5H9A7_HUMAN
K7EKZ5_HUMAN	J3KMX3_HUMAN	H0YM31_HUMAN	Q5JPC9_HUMAN
K7ERG9_HUMAN	J3KMY5_HUMAN	H0YMF1_HUMAN	Q5R211_HUMAN
K7ES70_HUMAN	J3KNQ2_HUMAN	H0YN42_HUMAN	Q5SR54_HUMAN
KNG1_HUMAN	J3KSK3_HUMAN	H3BQZ9_HUMAN	Q5STZ8_HUMAN
LAMC1_HUMAN	J3QRJ3_HUMAN	H3BRN4_HUMAN	Q5T025_HUMAN
LDHA_HUMAN	J3QSU6_HUMAN	H3BRS0_HUMAN	Q5T985_HUMAN
LEG1_HUMAN	K1C10_HUMAN	H3BS10_HUMAN	Q5URX0_HUMAN
LGMN_HUMAN	K22E_HUMAN	H3BTX9_HUMAN	Q5VY30_HUMAN
LRP1_HUMAN	K2C1_HUMAN	H7BYH4_HUMAN	Q96C32_HUMAN
LUM_HUMAN	K7EJB9_HUMAN	H7C402_HUMAN	Q96GW1_HUMAN

NS	M/R	IL-6/IL-6R	M/R/IL-6/IL-6R
LYAG_HUMAN	K7EKL3_HUMAN	H7C5R1_HUMAN	QSOX1_HUMAN
M0R2V7_HUMAN	K7EKZ5_HUMAN	H9KVB4_HUMAN	SAP3_HUMAN
M1BL1_HUMAN	K7ENT6_HUMAN	HBA_HUMAN	SFRP4_HUMAN
MASP1-4_HUMAN	K7EQQ3_HUMAN	HBB_HUMAN	SODE_HUMAN
MIF_HUMAN	K7ERG9_HUMAN	HCD2_HUMAN	SPRC_HUMAN
MMP2_HUMAN	KMT2C_HUMAN	HEMO_HUMAN	SRGN_HUMAN
NEGR1_HUMAN	KNG1_HUMAN	HEP2_HUMAN	STC2_HUMAN
NOTC2_HUMAN	LAMC1_HUMAN	HINT1_HUMAN	SYDE2_HUMAN
NOTC3_HUMAN	LDHA_HUMAN	HMCS2_HUMAN	THBG_HUMAN
NUCB1_HUMAN	LG3BP_HUMAN	HPT_HUMAN	TIMP2_HUMAN
PAI1_HUMAN	LGMN_HUMAN	HSDL2_HUMAN	TPIS_HUMAN
PCOC1_HUMAN	LRP1_HUMAN	IBP4_HUMAN	TPX2_HUMAN
PCP_HUMAN	LRP8_HUMAN	IBP6_HUMAN	TRFE_HUMAN
PEBP1_HUMAN	LUM_HUMAN	IBP7_HUMAN	TSP1_HUMAN
PEDF_HUMAN	MASP1-4_HUMAN	IC1_HUMAN	TTHY_HUMAN
PGBM_HUMAN	MFGM_HUMAN	IDHC_HUMAN	USP9Y_HUMAN
PGRP2_HUMAN	MIF_HUMAN	IDHP_HUMAN	VASN_HUMAN
PGS1_HUMAN	MMP2_HUMAN	IGKC_HUMAN	VATA_HUMAN
PGS2_HUMAN	NAA16_HUMAN	IGLC3_HUMAN	VTNC_HUMAN
PLBL2_HUMAN	NEGR1_HUMAN	INT11-4_HUMAN	X6R3G6_HUMAN
PLMN_HUMAN	NEUS_HUMAN	IPSP_HUMAN	ZA2G_HUMAN
PPARD_HUMAN	NP1L2_HUMAN	ISLR_HUMAN	ZFR_HUMAN
PPIB_HUMAN	NUCB1_HUMAN	J3KMX3_HUMAN	
PROF1_HUMAN	O60597_HUMAN	J3KMY5_HUMAN	
PROZ_HUMAN	PAI1_HUMAN	J3KND8_HUMAN	
PTGDS_HUMAN	PCOC1_HUMAN	J3KQ18_HUMAN	
PTX3_HUMAN	PCP_HUMAN	J3QSU6_HUMAN	
PZP_HUMAN	PEAK1_HUMAN	K1C10_HUMAN	
Q4VY20_HUMAN	PEBP1_HUMAN	K1C9_HUMAN	
Q5H9A7_HUMAN	PEDF_HUMAN	K22E_HUMAN	
Q5JPC9_HUMAN	PGBM_HUMAN	K2C1_HUMAN	
Q5R211_HUMAN	PGRP2_HUMAN	K2C6B_HUMAN	
Q5T123_HUMAN	PGS1_HUMAN	K7EJ44_HUMAN	
Q5T985_HUMAN	PGS2_HUMAN	K7ERG9_HUMAN	
Q5URX0_HUMAN	PI16_HUMAN	KIF4A_HUMAN	
Q5VY30_HUMAN	PLMN_HUMAN	KNG1_HUMAN	
Q6L983_HUMAN	PRELP_HUMAN	KPYR_HUMAN	
Q96C32_HUMAN	PROF1_HUMAN	LAMC1_HUMAN	
QSOX1_HUMAN	PROZ_HUMAN	LAMC2_HUMAN	
S4R460_HUMAN	PTGDS_HUMAN	LG3BP_HUMAN	

NS	M/R	IL-6/IL-6R	M/R/IL-6/IL-6R
SAHH2_HUMAN	PTX3_HUMAN	LMBD2_HUMAN	
SDC4_HUMAN	PZP_HUMAN	LRP8_HUMAN	
SFRP4_HUMAN	Q4VY20_HUMAN	LUM_HUMAN	
SODE_HUMAN	Q5H9A7_HUMAN	M0R0F0_HUMAN	
SOX6-2_HUMAN	Q5JPC9_HUMAN	M0R2B3_HUMAN	
SPRC_HUMAN	Q5JSG7_HUMAN	MEG10_HUMAN	
SRGN_HUMAN	Q5T123_HUMAN	MMP2_HUMAN	
STC2_HUMAN	Q5T321_HUMAN	MMSA_HUMAN	
TGON2_HUMAN	Q5T7F0_HUMAN	NTF3_HUMAN	
THBG_HUMAN	Q5T985_HUMAN	NUCB1_HUMAN	
THIO_HUMAN	Q5URX0_HUMAN	ODBB_HUMAN	
TIMP2_HUMAN	Q5VY30_HUMAN	PAI1_HUMAN	
TPIS_HUMAN	Q96C32_HUMAN	PCOC1_HUMAN	
TRFE_HUMAN	QSOX1_HUMAN	PDIA3_HUMAN	
TSP1_HUMAN	S4R460_HUMAN	PEDF_HUMAN	
TTHY_HUMAN	SAP3_HUMAN	PGBM_HUMAN	
TXND5_HUMAN	SDC4_HUMAN	PGRP2_HUMAN	
UBR3-2_HUMAN	SFRP4_HUMAN	PGS1_HUMAN	
V9GYS1_HUMAN	SHQ1_HUMAN	PGS2_HUMAN	
V9GZ57_HUMAN	SODE_HUMAN	PPARD_HUMAN	
VASN_HUMAN	SPRC_HUMAN	PPIB_HUMAN	
VILL_HUMAN	SRGN_HUMAN	PRDX3_HUMAN	
VINC_HUMAN	STC2_HUMAN	PRDX6_HUMAN	
VNN1_HUMAN	TGON2_HUMAN	PSB2_HUMAN	
VTNC_HUMAN	THBG_HUMAN	PTX3_HUMAN	
X6R3G6_HUMAN	THIO_HUMAN	Q4VY20_HUMAN	
ZA2G_HUMAN	TIE2_HUMAN	Q5H9A7_HUMAN	
ZC3H4_HUMAN	TIMP2_HUMAN	Q5JPC9_HUMAN	
	TPIS_HUMAN	Q5JR01_HUMAN	
	TRFE_HUMAN	Q5SR54_HUMAN	
	TSP1_HUMAN	Q5T025_HUMAN	
	TTHY_HUMAN	Q5T985_HUMAN	
	TWSG1_HUMAN	Q5URX0_HUMAN	
	TYB4_HUMAN	Q5VY30_HUMAN	
	V9GYM3_HUMAN	Q5VZC3_HUMAN	
	V9GZ57_HUMAN	Q5W0H4_HUMAN	
	VASN_HUMAN	Q8IUN7_HUMAN	
	VILL_HUMAN	Q96C32_HUMAN	
	VINC_HUMAN	QSOX1_HUMAN	
	VNN1_HUMAN	S4R3C6_HUMAN	

NS	M/R	IL-6/IL-6R	M/R/IL-6/IL-6R
	VWF_HUMAN	S4R3P0_HUMAN	
	WISP2_HUMAN	SFRP4_HUMAN	
	ZA2G_HUMAN	SLU7_HUMAN	
	ZC3H4_HUMAN	SNED1_HUMAN	
		SODE_HUMAN	
		SPRC_HUMAN	
		SPT2_HUMAN	
		SPYA_HUMAN	
		STC2_HUMAN	
		TGM2_HUMAN	
		THBG_HUMAN	
		THIL_HUMAN	
		THIO_HUMAN	
		TIG1_HUMAN	
		TIMP2_HUMAN	
		TPIS_HUMAN	
		TPX2_HUMAN	
		TRFE_HUMAN	
		TSP1_HUMAN	
		TTHY_HUMAN	
		TVBX1_HUMAN	
		U3KPR1_HUMAN	
		V9GYG0_HUMAN	
		V9HW50_HUMAN	
		VASN_HUMAN	
		VTNC_HUMAN	
		WDFY4_HUMAN	
		X6R3G6_HUMAN	
		X6RBG4_HUMAN	
		ZA2G_HUMAN	
		ZN638-3_HUMAN	

Table S3: Top 10 molecular network predicted by IPA for IL-6/IL-6R secretome

ID	Molecules in Network	Score	Focus Molecules	Top Diseases and Functions
1	20s proteasome, ACOT13, B2M, CRYZ, DECR1, ECM1, EEF1A1, EEF2, FN1, GAPDH, GOLGA4,HSP, Hsp70, IFN Beta, Ifnar, JAK, LGALS3BP, mediator, MHC CLASS I (family), MHC Class II (complex), PPIB, PSAP, PSMA2, PSMB2, PTGR1, RNA polymerase II, Rnr, RPS23, RPS5, SNED1, SOD1, SPTY2D1, TKT, TPT1, Ubiquitin	39	23	[Cancer, Organismal Injury and Abnormalities, Respiratory Disease]
2	7S NGF, AFM, Akt, ATRN, C1q, C1R, C1RL, C1S, CFI, Collagen type V, Collagen type VI, Complement, Complement component 1, CSPG, DCN, Hnf3, Igf, Igfbp, IGFBP4, IGFBP5, IGFBP6, IGFBP7, IGHG3, Iti, ITIH2, ITIH3, Laminin (complex), LRG1, MEGF10, PTX3, SERPING1, Smad2/3-Smad4, UMOD, VCAN, VitaminD3-VDR-RXR	32	20	[Dermatological Diseases and Conditions, Hereditary Disorder, Immunological Disease]
3	ABAT,ACAN,AGXT,AKR1C1/AKR1C2,BGN,COL12A1,COL1A2,COL3A1,COL6A1,COL6A2,COL6A3,collage, Collagen Alpha1,Collagen type I (complex), Collagen type III, collagenase, ERK, FBN1, Hepatic Transaminase, IL6R, JINK1/2, JUN/JUNB/JUND, N-cor, Nr1h, PCOLCE, Pdi, PEPCK, PPBP, Rar, Rxr, SERPINF1, SPARC, T3-TR-RXR, TGFBI, TIMP2	30	19	[Dermatological Diseases and Conditions, Inflammatory Disease, Organismal Injury and Abnormalities]
4	Ap1, Atrial Natriuretic Peptide, Cdc2, CLEC3B, CLU, CP, Cpla2, Cytokeratin, FTCD, ISLR, Keratin, KNG1, KRT1, KRT18, KRT2, KRT6B, KRT8, KRT86, KRT9, LRP, LRP8, LTF, MIR124, Na, K-ATPase, PCYOX1, PI3K (complex), plasminogen activator, Pmca, PRKAA, PROTEASE, SERPINE1, TLR2/TLR4, VIM, Vla-4, VTN	30	19	[Cell Morphology, Embryonic Development, Hair and Skin Development and Function]
5	A1BG,Adaptor protein 1, AHSG, AMBP, APOH, BHMT, C4A/C4B, creatine kinase, CSF, ERK1/2, Ferritin, HDL, HDL-cholesterol, hemoglobin, Hif, HP, HPX, HSPG2, IL-1R, IL1RL1,INTERLEUKIN, KLKB1, LCN, ORM1, ORM2, PAEP, PDGF (family), RBP4, SAA, SERPINC1, SERPINF2, Stat3-Stat3, Tcf 1/3/4, TTR, VLDL	28	18	[Cellular Compromise, Inflammatory Response, Neurological Disease]
6	aldehyde dehydrogenase, aldehyde dehydrogenase (NAD), ALDH, ALDH1A1, ALDH1L1, ALDH2, ALDH4A1, ALDH6A1, ALDOB, Aldose Reductase, ALT, B-cell receptor, BRMS1L, FBP1, Fc gamma receptor, Fcgr3, HEXB, HNF4α dimer, Iga, IgG, IgG1, IgG2a, Igg3, IGHA1, IGHA2, IGHG1, IGHG4, IGKC, Jnk, KRT10, PBLD, PCK2, Serpin, TF, transglutaminase	28	18	[Humoral Immune Response, Inflammatory Response, Small Molecule Biochemistry]
7	3-hydroxyacyl-CoA dehydrogenase, ACAT1, ADH1A, ADH1B, ADH4, ADH5, ADH6, alcohol dehydrogenase, ASS1, C/EBP, caspase, CDK4/6, Cyclin A, Cyclin D, Cyclin E, cytochrome C, cytochrome-c oxidase, E2f, EGLN, GLUD1, HADH, HMGCS2, HSD17B10, HSD17B4, MAOB, Mitochondrial complex 1, MTHFD1, Pkc(s), PRDX3, Rb, Rho gdi, SELENBP1, STC2, TCF/LEF, TPI1	28	18	[Energy Production, Lipid Metabolism, Small Molecule Biochemistry]
8	aldo,Alpha 1 antitrypsin, Apolipoprotein, C4, C4BP, Cathepsin, Ces, CES1, CFD, Collagen type II, Collagen type ix, CST3, CTSB, CTSD, CTSL, DDT, Gpd, GPD1, IL6,	26	17	[Cellular Compromise, Post-Translational

	Kallikrein, LUM, Lysosomal Protease, MIRLET7, Neuro-filament, RARRES1, Rbp, RIDA, Serine Protease, SERPINA3, SERPINA5, SERPINA7, SERPIND1, Timp, TPP1, trypsin			Modification, Protein Degradation]
**9**	ACAC, Ap2, APOA1, APOB, APRT, ATP synthase, AZGP1, C3, CAT, CLSTN1, CPT1, EEF1A, EFEMP1, FABP1, FH, glutathione peroxidase, HSDL2 ,HYOU1, Ldh (complex), LDL, LDL-cholesterol, MAC, MPST ,Nfat (family), NPC2, P38 MAPK, PI3K (family), SLC6A2, SMAD1/5/9, Sod, SOD2, SOD3, SYK/ZAP, TH2 Cytokine, VLDL-cholesterol	26	17	[Organismal Injury and Abnormalities, Renal Damage, Renal Tubule Injury]
**10**	ADRB, Angiotensin II receptor type 1, ANXA5, CPS1, DKK3, Fcer1, G protein, GST, GSTA3, HBB, HINT1, IDH1, IDH2, Ikb, LMBRD2, MAP2K1/2, MHC Class I (complex), MTORC2, NADPH oxidase, NFAT (complex), NFkB (family), Notch, Pak, PDIA3, peroxidase (miscellaneous), POLG, PPIA, PRDX2, PRDX6, QSOX1, Sapk, Secretase gamma, UGP2, VASN, Vegf	26	17	[Cancer, Endocrine System Disorders, Organismal Injury and Abnormalities]

SUPPLEMENTARY FIGURES

Figure S1: (A) Culture of bone marrow derived macrophages from C56BL/6 mice with IL-6+IL-6R for 12 day (B) IL-6+IL-6R along with M+R cotreatment shows an increased number of TRAP-positive osteoclast progenitor cells (n=3; $p<0.05$). Representative image acquired using 40X and the size of scale is 300 µm.

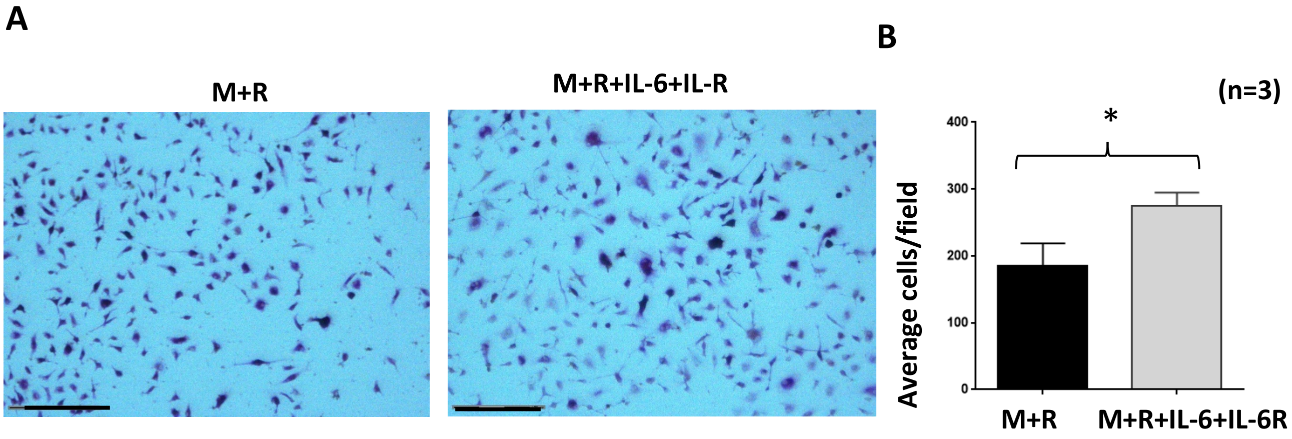

Figure S2: CD14 positive cells were shorted using anti-CD14 antibody (mouse monoclonal) on human THP1 monocytic cell line as well as on 3 independent human patients derived RASFs using Alexa Fluor anti mouse 488 antibody. All 3 human RASF were more than passage 5. Human THP1 cell line were found to be enriched with CD14+ cells for more than 17% cell populations. While human RASFs subjects had less than 1% to no stating for CD14 antibodies. Experimental RASF which is grown more than passage 5 are devoid of any visible contamination of CD14+ cells.

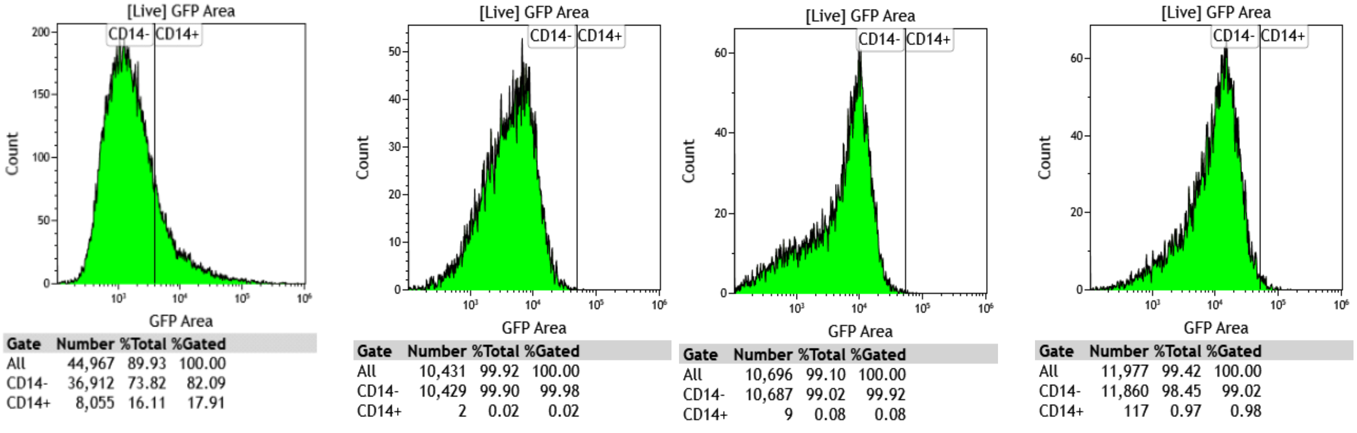

THP1 monocytes

RASF patient 1
Passage 6

RASF patient 2
Passage 5

RASF patient 3
Passage 6

Figure S3: Gene Ontology study of affected biological function from RASFs were analyzed using metascape.org gene ontology platform. **S1A)** Human RASFs stimulated with M-CSF/RANKL with 261 secreted proteins; **S1B)** Human RASFs stimulated with IL-6/IL-6R with 288 proteins and **S1C)** Human RASFs stimulated M-CSF/RANKL/IL-6/IL-6R (All) with 199 secretory proteins.

S3A)

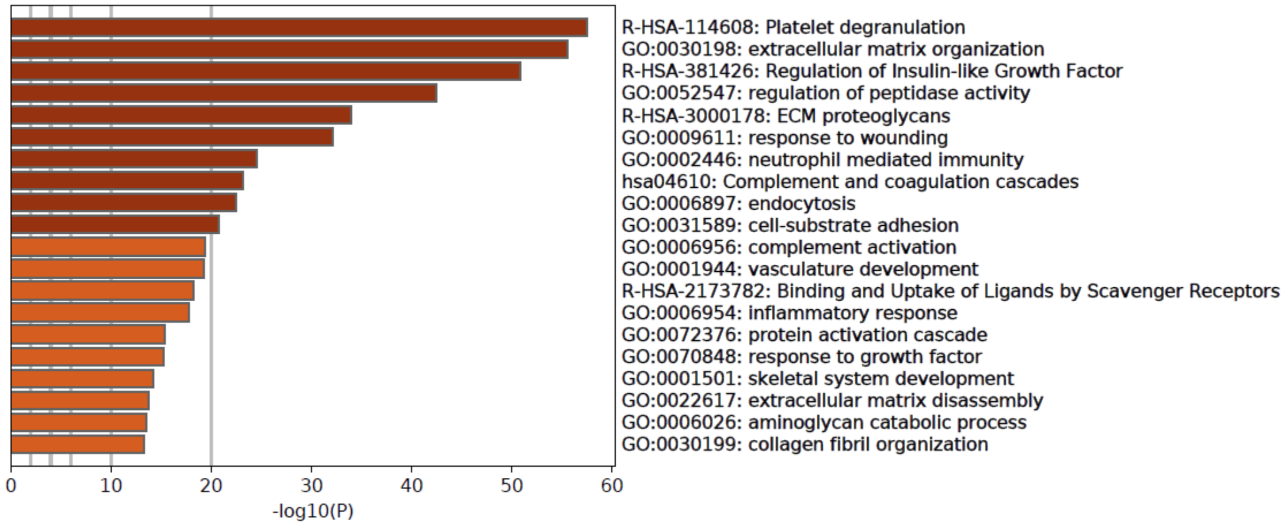

S3B)

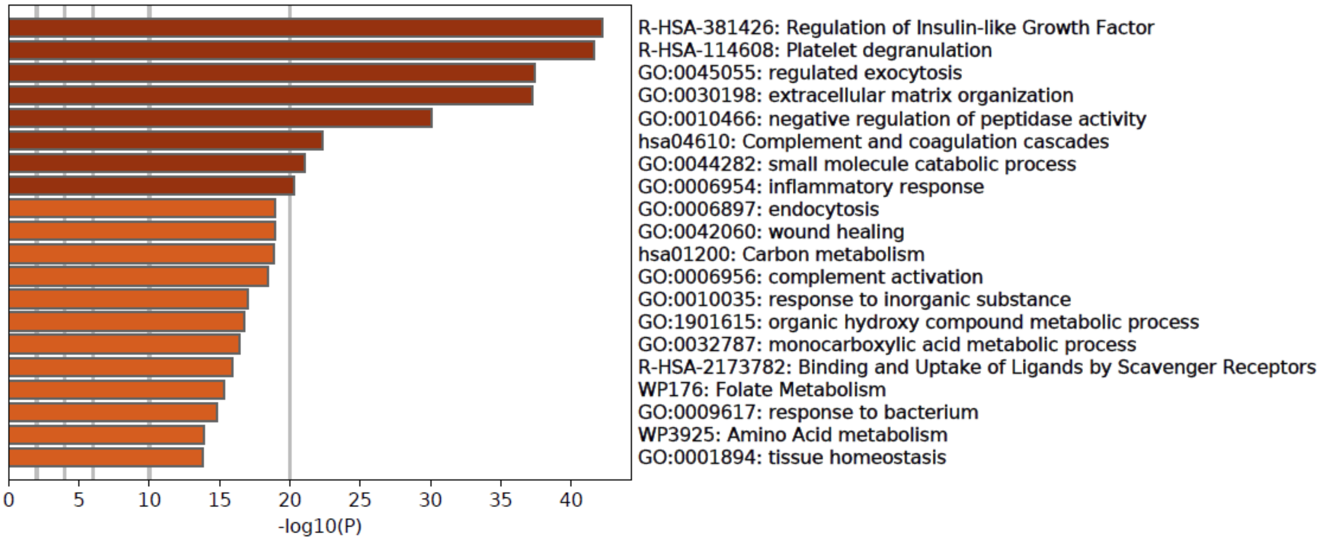

S3C)

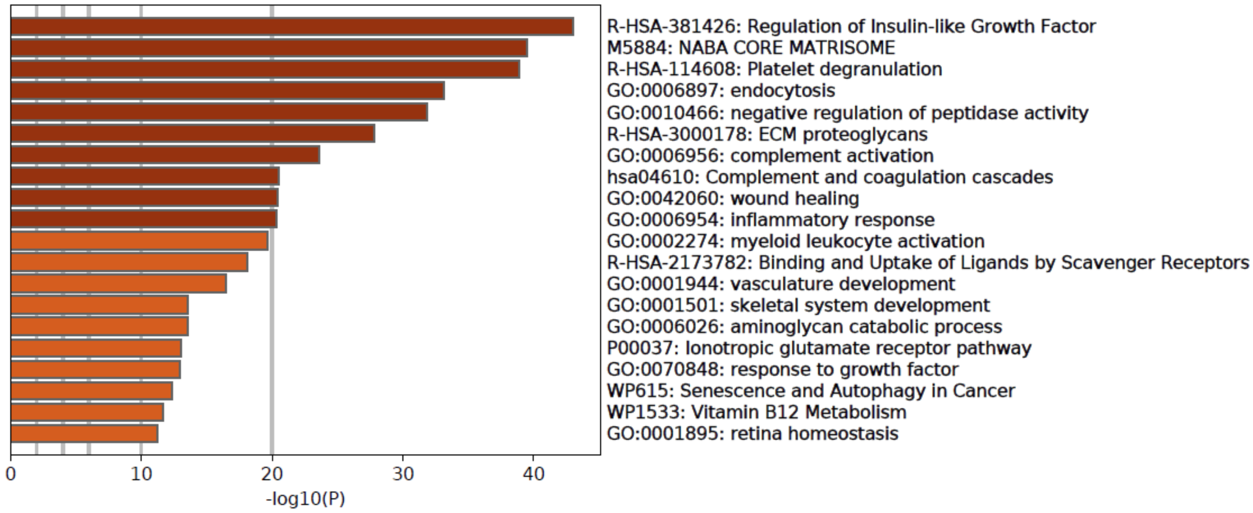

Figure S4 : Nuclear and cytoplasmic extract were evaluated for presence of Tubulin and Lamin B presence from NS, MR, IL-6+IL-6R, MR+IL-6+IL-6R and MR+IL-6+IL-6R+tofacitinib treated samples (in main figure 5.A). We observed no cross contamination of Tubulin and or Lamin B in different cellular fractions.

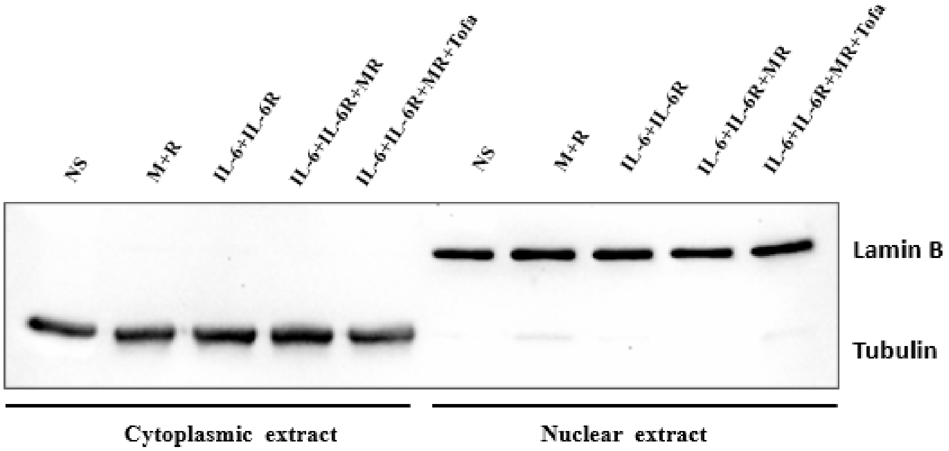

Supplement: Supplementary file 1 [file DataSheet_1.pdf]
